# Genetic analysis of ear, husk, and tassel traits in tropical maize under diverse environments

**DOI:** 10.3389/fpls.2025.1618054

**Published:** 2025-08-11

**Authors:** Titus Kosgei, Dan Makumbi, Edna K. Mageto, Hilda M. Kavai, George O. Ochieng, Carolyne A. Adhiambo, Joseph S. Kasango, Joan J. C. Kimutai, Rachael M. Kamau, Julius P. Sserumaga

**Affiliations:** ^1^ International Maize and Wheat Improvement Center (CIMMYT), Nairobi, Kenya; ^2^ Independent Consultant, Ames, IA, United States; ^3^ National Livestock Resources Research Institute, National Agricultural Research Organization, Kampala, Uganda

**Keywords:** combining ability, ear length, ear circumference, heritability, heterosis, path analysis, tassel traits, *Striga hermonthica*

## Abstract

Grain yield (GY) in maize (*Zea mays* L.) is influenced by multiple component traits, with ear- and tassel-related traits playing a significant role. Despite their importance, these traits receive less emphasis in tropical maize breeding. This study aimed to: (i) assess the inheritance and heterosis of ear and tassel traits, and (ii) investigate their genetic correlation with GY. Thirty tropical maize inbred lines were used to develop 150 hybrids, which were evaluated under artificial *Striga hermonthica* infestation, managed drought stress, and rainfed conditions over two years. General (GCA) and specific (SCA) combining ability mean squares were significant (*P*< 0.05) for most traits, indicating the contribution of both additive and nonadditive gene action. GCA sums of squares predominated over SCA, suggesting that additive gene action controlled the inheritance of ear, husk, and tassel traits. Broad-sense heritability was high for husk (*H^2^* = 0.59–0.89), ear (*H^2^* = 0.71–0.93), and tassel (*H^2^* = 0.78–0.95) traits. Fifteen inbred lines exhibited significant positive GCA effects for ear traits, suggesting the presence of favorable alleles associated with increased ear length (ERL) and circumference (ERC). Additionally, 23 inbred lines exhibited favorable GCA effects for reduced tassel size. Mid-parent heterosis for ERL (41%) and ERC (22%) was greater under managed drought stress. Both ERL and ERC were strongly correlated with GY (*r*_g_ = 0.58–0.96), suggesting their suitability for inclusion in a selection index. Path analysis identified ERL and ERC as having significant positive direct effects on GY, while ear aspect had a negative direct effect on GY across all conditions. Selection for ear and tassel traits in tropical maize is recommended to develop more efficient inbred lines for higher grain yield.

## Introduction

Maize (*Zea mays* L.) contributes significantly to caloric intake and livelihoods in sub-Saharan Africa (SSA). Projections indicate that maize demand will grow at an annual rate of approximately 1.3%, largely driven by its versatility in food, feed, and industrial applications (Ranum et al., 2014). To meet current and future demands, maize productivity per unit area for maize must increase (Mansfield and Mumm, 2014). In SSA, maize yield averages approximately 2.0 t ha^-1^, while the global average is about 5.8 t ha^-1^ (Erenstein et al., 2022). More recent estimates indicate an average maize yield of 2.2 t ha^-1^ in 2023 in SSA (FAOSTAT, 2024). The low maize yields in SSA are attributed to various factors: biotic and abiotic stresses, limited use of inputs, poor agronomy, limited availability of certified hybrid seed, and the prevalence of old maize varieties (Bänziger et al., 2006; Mahuku et al., 2015; Abate et al., 2017; Cairns et al., 2021). Achieving significant maize yield gains, particularly through the development and dissemination of multiple stress-tolerant varieties, is essential for improving maize productivity in SSA. Furthermore, the development of stress tolerant varieties must consider maize genotypes with optimal plant architecture that efficiently utilize assimilates.

The development of efficient plant types using yield components and improved morphological and other physiological traits has been suggested as a key component in crop improvement strategies (Donald, 1968). In many regions, maize yield gains have been attributed to breeding, the use of hybrids, improved crop management practices, and increased plant density (Duvick, 2005; Fasoula and Fasoula, 2002; Lee and Tollenaar, 2007). The relationship between grain yield and morphological characteristics, specifically the process of assimilate production (source), and dry matter accumulation (sink) is critical and a fundamental determinant of maize grain yield (Lee and Tollenaar, 2007; Egli, 2015). Key source traits linked to increased maize productivity include smaller tassels, erect leaves, increased stay-green characteristics during kernel filling, better radiation use efficiency, slower leaf senescence, longer kernel filling periods, reduced lodging, and improved disease and insect resistance (Cavalieri and Smith, 1985; Duvick et al., 2004; Duvick, 2005; Tollenaar and Aguilera, 1992). Sink traits such as ear size (ear length and diameter), ears per plant, kernel number, kernel size, kernel rows per ear have also been associated with higher yield (for a review, see Egli, 2015).

Breeding for reduced tassel size in maize has the potential to enhance photosynthetic efficiency and improve grain yield. Smaller tassels require fewer resources for development, allowing for greater allocation of photosynthetic assimilates and energy towards grain filling (Grogan, 1956; Duncan et al., 1967). Sangoi (2001) reported a 20% reduction in allocation of assimilates to the tassel in improved hybrids. Large tassels reduce light interception in the upper maize canopy (Grogan, 1956), and studies have shown negative correlations between tassel branch numbers and grain yield (Geraldi et al., 1985). Lambert and Johnson (1978) noted that increased grain yield in case of reduced tassel branches results from reduced shading effects in the maize canopy. Lauer et al. (2012) reported a downward trend in tassel branch numbers in temperate maize inbred lines from the 1930s to the 2000s.

Maize ear length and circumference are key morphological traits determining maize grain yield (Huo et al., 2016), with the ear serving as a significant storage reservoir for photosynthetic assimilates. Environmental stress can affect the expression of ear length, leading to variability across different growing conditions (Nielsen, 2003). Maize ear husks are important photosynthetic organs of maize, with high carbon assimilation efficiency and a significant contribution to kernel dry matter per unit area (Fujita et al., 1995). Husk traits such as husk width, husk length, and husk number are important for mechanical harvesting of maize (Zhou et al., 2020), with fewer husks being more conducive for mechanical harvesting (Troyer and Ambrose, 1971). Studies on temperate and other maize germplasm have highlighted the presence of genetic correlations among husk, ear, and tassel traits tropical germplasm (Zhou et al., 2020).

Understanding of the variability and inheritance mechanisms of important source-sink traits in maize is crucial for developing improved high-yield, efficient maize varieties. Combining ability studies have reported the importance of additive gene action over non-additive gene action for ear traits (Mock and Schuetz, 1974; Han and Hallauer, 1989; Fan et al., 2008), husk traits (Brewbaker and Kim, 1979), and tassel branch number (Betrán and Hallauer, 1996; Brewbaker, 2015) in temperate maize. However, in tropical maize, ear length, diameter, and circumference were under the influence of both additive and nonadditive effects (Dhillon and Singh, 1977; Kamara et al., 2020). While evidence of genetic improvements in key agronomic traits associated with increased grain yield, such as ear and tassel characteristics, is available for temperate maize (Duvick et al., 2004, Duvick, 2005; Lauer et al., 2012), comparable data for tropical maize is limited.

Few studies have investigated the genetics of ear or tassel traits in tropical maize, with research limited to one to two traits under optimal management conditions (Kamara et al., 2014; Nardino et al., 2016; Kamara et al., 2020; Onejeme et al., 2020). None of these studies have investigated ear, husk, and tassel traits in a large set of African-adapted tropical maize inbred lines under managed stress environments in a single study. The objectives of this study were to: (i) investigate the inheritance of ear, and tassel traits among tropical maize inbred lines and estimate the heterosis, and (ii) examine genetic correlations between grain yield and other yield component traits under contrasting management conditions.

## Materials and methods

### Genetic materials

This study used 30 inbred lines of diverse origins and breeding history, including 25 lines from the International Maize and Wheat Improvement Center (CIMMYT) and five *Striga*-resistant lines from the International Institute of Tropical Agriculture (IITA) (**Supplementary Table S1**). The CIMMYT lines included 11 doubled haploid (DH) lines developed from F_2_ populations and 10 lines developed through pedigree breeding. Additionally, four elite CIMMYT lines (CML312, CML543, CML610A, and CKL12128), which express varying levels of drought tolerance were included in the study. The 30 inbred lines were grouped into six sets, with each set having five inbred lines. Lines from one set (females) were crossed with lines from another set (males) in a North Carolina Design II (NC II) mating scheme (Comstock and Robinson, 1948) resulting in 150 experimental single-cross hybrids. Each inbred line was used once as either a female or male in different sets. We used the NC II mating design with sets to reduce the number of hybrids generated when crossing many inbred lines. More details about the NCII with sets design and its use can be found in Hallauer and Miranda Filho (1988, p. 67–72).

### Experimental design, test locations

#### Hybrid trials

The 150 single-cross hybrids, along with two internal genetic gain and four commercial checks were grown in nine trials planted at five locations in Kenya in 2020 and 2021 (**Supplementary Table S2**). The experimental design was a 4 × 39 alpha-lattice design (Patterson and Williams, 1976), with two replications. Each experimental unit consisted of one row 4 m long, spaced 0.75 m between rows and 0.20 m between plants, to give a final plant population density of approximately 66,666 plants ha^-1^ at all locations. The 156 hybrids were evaluated in five field trials under artificial *Striga* infestation at the Kenya Agricultural and Livestock Research Organization (KALRO) research stations at Kibos and Alupe, and at Siaya ATC. Two hybrid trials were planted under rainfed conditions at KALRO Kakamega. Two managed drought stress trials were planted at KALRO Kiboko Research Center. All hybrids were tested in every environment. The location characteristics and soil types at the five locations have been described previously by Makumbi et al. (2015; 2018) and Kimutai et al. (2024). Standard agronomic and cultural practices were followed as recommended for each location.

#### Line trials

A line evaluation trial, comprising of the 30 parental lines of the 150 experimental hybrids, was formed and laid out as a 3 × 10 alpha-lattice design with two replications. Each experimental unit consisted of two 5 m long rows, spaced 0.75 m between rows and 0.25 m between plants. The inbred line trials were evaluated at the same locations and conditions as the hybrid trials, with the line trials planted side by side with the hybrid trial at all sites. In total, nine inbred line trials were conducted, including five under artificial *Striga* infestation, two under rainfed conditions, and two under managed drought stress. Standard agronomic and cultural practices, as recommended for each location, were followed.

### Artificial *Striga* infestation and managed drought stress

Artificial *Striga hermonthica* infestation (hereafter referred to as *Striga*) was used to ensure uniform exposure to *Striga* for all genotypes at three locations: Alupe, Kibos, and Siaya ATC. The fields at Kibos and Alupe research had been previously used for imazapyr herbicide studies (Makumbi et al., 2015; Kanampiu et al., 2018). Due to the residual toxicity of imazapyr (Alister and Kogan, 2005), any *Striga* seeds present in the soil from prior experiments is killed. *Striga* inoculum was prepared and applied following protocols detailed by Makumbi et al. (2015) and Kanampiu et al. (2018). Di-ammonium phosphate (DAP, 18:46:0) fertilizer was applied at half the recommended rate (30 kg ha^-1^) at planting to promote plant establishment without suppressing *Striga* germination. A half dose (30 kg ha^-1^) of calcium ammonium nitrate (CAN, 26%) fertilizer was applied for topdressing at 4 weeks after planting. Hand weeding was conducted to remove all weeds except *Striga* plants. The managed drought stress trials were carried out under irrigated conditions during the rain-free period (June–October) at Kiboko. Irrigation water was applied using sprinklers and drip lines at planting to establish a good plant stand, with regular watering during vegetative growth to prevent water stress. During the study period, the average minimum and maximum temperature were 13.3°C and 32.7°C in 2020, and 13.6°C and 31.5°C in 2021. Irrigation water in the drought trial was withdrawn 45 days (V15 stage) after planting. Detailed drought stress management procedures are described in the manual by Bänziger et al. (2000). The anthesis-silking interval (ASI) was regularly calculated to determine any need for additional irrigation water during or after flowering. The average ASI for the hybrids and parental inbred lines was 0.9 and 2.4 days, respectively – both within the acceptable range for good drought stress management, as outlined by Bänziger et al. (2000). As a result, no further irrigation was necessary in the trials. Standard agronomic and cultural practices were followed as recommended for drought stress trials.

### Data collection

Data were recorded on agronomic, ear traits, and tassel on plot basis. The agronomic traits recorded included plant aspect, ear rot, and ear weight. Plant aspect was recorded on a scale of 1 to 5, where 1 indicated excellent and 5 indicated poor plant type. Bad husk cover was measured as percentage of plants with ears that are not completely covered by the husks. Ear aspect was recorded on a scale of 1 to 5, based on proper grain filling, ear uniformity, and ear rot infection, with 1 = uniform, large well-filled and clean ears, and 5 = ears with undesirable characteristics. Ear rot was recorded as percentage of harvested ears that are affected due to a combined effect of various fungal diseases. Ear weight was used to calculate grain yield (GY) expressed in t ha^-1^, adjusted to 80% shelling percentage and 12.5% grain moisture content. Husk traits (husk length, HSL; husk width, HSW, and husk number, HSN), and ear traits (ear length, ERL, and ear circumference, ERC) were measured. Husk traits were phenotyped at harvest following the method outlined by Cui et al. (2016). In brief, husk number was recorded from the outermost layer to the innermost layer, while husk length and width were measured on the 3^rd^ husk from the outside. Husk width was measured at the midpoint of the 3^rd^ husk. Ear length was measured from the base to the tip on eight cobs of well‐bordered plants, and ear circumference was measured on the same cobs. Data on ear traits was recorded on eight plants in a plot. Tassel branch number (TBN) and tassel branch length (TBL), were recorded by counting the number of tassel branches and measuring the branch length on 10 plants in a plot.

### Statistical analyses

#### Analysis of variance

All data were tested for normality using the Shapiro-Wilk test (Shapiro and Wilk, 1965) before analysis of variance. Analyses of variance were performed using PROC MIXED of SAS (SAS Institute, 2016). Entries were considered fixed effects while locations were considered random effects. The linear model below was used for combined analysis across each environment:


$$Y_{ijrk}=\mu +\alpha_{i}+\beta_{j}+\rho_{r}\left(\beta_{j}\right)+\lambda_{k}\left[\rho_{r}\left(\beta_{j}\right)\right]+\alpha \beta_{ij}+\epsilon_{ijrk}$$

where Y*_ijrk_* is the mean of the *i*th genotype, in the *r*th replicate within the *k*th subblock of the *j*th environment; μ is the grand mean; α*_i_* is the effect of the *i*th genotype; β*_j_* is the effect of the *j*th environment; ρ*_r_* is the effect of the *r*th replicate; ρ*_r_* (β*_j_*) is the effect of the replicates within environments; λ*_k_* [ρ*_r_* (β*_j_*)] is the effect of the incomplete blocks within replicates and environments; αβ*_ij_* is the effect of genotype × environment interaction; and ϵ*_ijrk_* is the residual error.

In the across-environment analysis of variance, the significance of the genotype effects was tested using the corresponding genotype × environment interaction as the error term, while the genotype × environment interaction was tested using the pooled error. Each location-year combination was considered a separate environment. All factors were considered random effects to estimate variance components. The best linear unbiased estimates (BLUEs) and the best linear unbiased predictions (BLUPs) were computed using META-R (Alvarado et al., 2020).

Broad-sense heritability was estimated for combined environments according to Hallauer et al. (2010) as:


$$H^{2}=\frac{\sigma_{G}^{2}}{\sigma_{G}^{2}+\frac{\sigma_{GE}^{2}}{e}+\frac{\sigma_{E}^{2}}{er}}$$

where $\sigma_{G}^{2}$ is the genotypic variance, $\sigma_{GE}^{2}$ is the variance of the interaction between the genotype and environment, e is the number of environments, r is the number of replicates, and the $\sigma_{E}^{2}$ is the residual variance.

Genotypic correlations were estimated using META-R for pairs of traits following Holland (2006) as:


$${\hat{r}}_{gij}=\frac{{\hat{\sigma }}_{gij}}{{\hat{\sigma }}_{gi}\sigma_{gj}}$$

where ${\hat{\sigma }}_{gij}$ is the estimated genotypic covariance between traits *i* and *j*, ${\hat{\sigma }}_{gi}$ and ${\hat{\sigma }}_{gj}$ are the estimated genotypic standard deviations for traits *i* and *j*, respectively.

Mid-parent (MPH) and high-parent (HPH) heterosis of all traits were calculated using the BLUEs of the hybrids and inbred lines. Mid-parent heterosis was calculated as $\text{MPH}=\frac{\left(F_{1}-\text{MP}\right)}{\text{MP}}\times 100$ where, F_1_ is the hybrid mean performance, and MP = (P_1_ + P_2_)/2 where P_1_ and P_2_ are the means of the two parents. High-parent heterosis was calculated as $\text{HPH}=\frac{\left(F_{1}-\text{HP}\right)}{\text{HP}}\times 100$ where HP is mean of the best parent.

#### Design II analysis

To estimate combining ability of the lines and hybrids, an analysis of variance was conducted for the 150 experimental hybrids using the PROC GLM of SAS (SAS, 2016) following the North Carolina Design II model (Comstock and Robinson, 1948). The following general linear model was used for the analysis across environments.


$$Y_{ijkl}=µ+m_{i}+f_{j}+{\left(m\times f\right)}_{ij}+{\left(m\times e\right)}_{ik}+{\left(f\times e\right)}_{jk}+{\left(m\times f\times e\right)}_{ijk}+e_{k}+r_{l}(e_{k})+\epsilon_{ijkl}$$

where Y*_ijkl_* is the observed trait value, *µ* is the grand mean, *m_i_* is the effect of the *i*th male, *f_j_* is the effect of the *j*th female, (*m × f)_ij_* is the effect of interaction between *i*th male and *j*th female, (*m × e)_ik_* is the effect of the *i*th male in the *k*th environment, (*f × e)_jk_* is the effect of the *j*th female in the *k*th environment, (*m × f × e)_ijk_* is the interaction effect between *i*th male and *j*th female in the *k*th environment, *e_k_* is the effect of the *k*th environment, *r_l_(e_k_)* is the effect of *l*th replication in the kth environment, and ϵ*_ijkl_* is the residual error.

In an NC II sets design, the variance components of variance for hybrids within sets are partitioned into those attributable to male (sets), female (sets), and the female × male (sets) interaction (Hallauer et al., 2010). The proportion of GCA-male, GCA-female, and SCA for each trait was computed as a percentage of the sum of squares for the hybrids in each environment. Estimates of GCA effects for agronomic, tassel, and ear traits for the inbred lines and SCA effects for each hybrid, were computed from BLUEs across environments using PROC MEANS of SAS (SAS, 2016).

#### Path analysis

To examine cause and effect relationships among grain yield, tassel, ear, and agronomic traits, BLUPs for these traits were subjected to sequential path analysis to mitigate against multicollinearity which occurs when two or more independent variables in a regression model are highly correlated (Samonte et al., 1998; Sserumaga et al., 2020). In sequential path analysis, traits were classified into first, second, or third order (or higher) based on their impact on the total variation in grain yield, using stepwise regression. Path coefficient analysis was performed separately for artificial *Striga* infestation and rainfed conditions, and a combination of both using SPSS version 20 (IBM, 2011).

## Results

### Analysis of variance

The combined ANOVA for the hybrids revealed significant (*P*< 0.01) mean squares for environment (E) and genotype (G) across most ear, tassel, and plant aspect traits under artificial *Striga* infestation, rainfed, and managed drought stress conditions (**Tables 1**, **2**). However, the genotype effect was not significant for ear rot (EROT) under artificial *Striga* infestation, and the environment effect was not significant for bad husk cover (BHC) under managed drought stress conditions. Partition of the genotype source of variation showed significant (*P*< 0.05) mean squares for GCA_m_/sets, GCA_f_/sets and SCA/sets for all traits under artificial *Striga* infestation except SCA/sets for EROT (**Table 1**). Under rainfed conditions, GCA_m_/sets, GCA_f_/sets and SCA/sets mean squares were highly significant (*P*< 0.001) for all traits except GCA_f_/sets for BHC, and SCA/sets for HSL, HSW, BHC, and tassel branch length (TBL) (**Table 2**). Under managed drought stress, GCA_m_/sets, GCA_f_/sets and SCA/sets mean squares were significant (*P*< 0.05) for all traits except GCA_m_/sets for BHC, and SCA/sets for HSW.

**Table 1:** Mean squares from combined ANOVA for husk, ear, tassel, and plant aspect traits of 150 NCII hybrids evaluated under artificial *Striga* infestation at three locations in 2020 and 2021.

Source	df	HSL^a^	HSW	HSN	ERC	ERL	EROT	df	BHC	EASP	TBL	TBN	df	PASP
Environment (E)	4	301.01***	90.84***	192.07***	182.15***	414.16***	107.50**	3	87177.82***	23.38***	35.83***	159.77***	1	9.75***
Sets	5	40.66***	12.69***	25.02***	44.10***	222.51***	35.30	5	23689.05***	3.66***	94.87***	245.03***	5	0.85***
E × sets	20	1.52	1.41	1.16	0.67	4.18**	26.01	15	6369.77***	0.31	9.70***	13.83**	5	0.08
Rep (E × sets)	30	2.30	2.97***	2.41**	0.67	8.94***	59.35***	24	186.09*	0.32*	3.99***	6.63	12	0.19
Genotype	149	14.18***	6.68***	7.20***	4.54***	17.15***	23.97	149	1499.92***	0.73***	15.91***	93.92***	149	0.21***
GCA_m_ /sets	24	33.80***	13.09***	15.51***	7.77***	19.09***	35.42**	24	1820.82***	1.24***	32.72***	243.91***	24	0.20*
GCA_f_ /sets	24	29.55***	14.54***	15.45***	5.34***	24.27***	28.86*	24	1389.69***	1.14***	36.18***	240.89***	24	0.24**
SCA/sets	96	4.03***	2.78***	2.13***	1.46***	4.16***	19.30	96	291.57***	0.34***	2.53***	11.82***	96	0.18*
E × Genotype	600	3.94***	2.05***	2.43***	1.77***	4.96***	21.16	450	1075.36***	0.37***	2.01***	6.98**	150	0.20***
E × GCA_m_ /sets	96	2.12	1.47	1.34*	0.60	1.58	20.12	72	539.23***	0.20	2.26***	5.99	24	0.10
E × GCA_f_ /sets	96	2.21	1.66	1.13	0.62	2.61*	16.13	72	418.99***	0.21	2.04***	5.70	24	0.29***
E × SCA/sets	384	1.85	1.40	1.12*	0.53	2.18	21.53*	288	200.83***	0.22	1.18	5.60	96	0.11
Pooled Error	718	1.76	1.39	0.96	0.50	2.00	17.98	573	109.69	0.19	1.10	5.61	288	0.12

*, **, *** Significant at the 0.05, 0.01, and 0.001 probability levels, respectively.

a BHC, Bad husk cover; EASP, Ear aspect; ERL, Ear length; ERC, Ear circumference; EROT, Percentage of rotten ears; HSL, Husk length; HSN, Husk number; HSW, Husk width; PASP, Plant aspect; TBL, Tassel branch length; TBN, Tassel branch number.

**Table 2:** Mean squares from combined ANOVA for husk, ear, tassel, and plant aspect traits of 150 NCII hybrids evaluated under rainfed conditions at Kakamega and managed drought stress at Kiboko in 2020 and 2021.

Source	df	HSL^a^	HSW	HSN	ERC	ERL	BHC	EASP	EROT	TBL	TBN	PASP
Rainfed conditions												
Environment (E)	1	3.47	3.77	233.01***	71.80***	282.90***	8337.97***	8.79***	39496.36***	154.53***	2233.39***	1.17**
Sets	5	26.60***	19.75***	5.09**	56.95***	222.31***	7901.70***	11.68***	1457.20***	25.87***	140.57***	3.60***
E × sets	5	6.44***	4.78*	2.83	2.07***	3.56***	493.33	1.92***	407.36*	4.58	19.59*	0.28
Rep (E × sets)	12	3.56**	7.32***	3.34*	0.45	1.52*	166.46	0.39***	1516.21***	4.49	6.89	0.15
Genotype	149	6.60***	4.34***	4.38***	3.59***	12.67***	847.42***	0.80***	331.46***	8.70***	41.73***	0.43***
GCA_m_ /sets	24	16.86***	8.31***	6.31***	4.17***	11.68***	1277.98***	0.87***	517.91***	18.83***	80.99***	0.38***
GCA_f_ /sets	24	11.79***	6.88***	8.27***	3.25***	11.65***	420.08	0.86***	408.07***	19.92***	103.46***	0.56***
SCA/sets	96	1.68	1.92	2.81***	0.75***	2.24***	474.20	0.20***	204.15**	2.47	11.34***	0.25**
E × Genotype	150	1.66	1.48	3.31***	1.14***	3.26***	341.59	0.27***	426.13	3.61**	24.31***	0.21*
E × GCA_m_ /sets	24	2.11*	1.15	1.54	0.61*	1.30	276.89	0.10	122.35	2.46	13.5**	0.17
E × GCA_f_ /sets	24	2.46**	1.85	1.39	0.59*	1.70**	146.27	0.28***	225.12*	3.83	12.98**	0.33**
E × SCA/sets	96	1.08	1.30	1.81	0.60***	1.18*	312.50	0.13	143.12	2.23	7.07	0.17
Pooled Error	288	1.31	1.79	1.64	0.37	0.84	304.90	0.12	137.35	2.53	6.93	0.16
Managed drought stress												
Environments (E)	1	46.25***	152.61***	38.03***	7995.57***	16.14**	0.02	2.67**	–	–	–	–
Sets	5	26.62***	4.84***	10.56***	7.15***	63.15***	200.17*	4.02***	–	–	–	–
E × sets	5	0.74	1.00	2.96**	1.30***	5.30**	86.57	0.54	–	–	–	–
Rep (E × sets)	12	2.50	4.23***	1.45	0.36	2.47	189.77**	0.67**	–	–	–	–
Genotype	149	7.25***	2.24***	4.65***	1.07***	7.35***	132.10**	0.65***	–	–	–	–
GCA_m_ /sets	24	13.24***	3.67***	10.24***	1.38***	10.32***	101.51	0.69***	–	–	–	–
GCA_f_ /sets	24	9.42***	4.35***	10.69***	1.65***	8.72***	118.83*	0.98***	–	–	–	–
SCA/sets	96	4.21***	1.23	1.43***	0.53***	3.36***	109.00**	0.38*	–	–	–	–
E × Genotype	150	2.730*	1.91***	1.20*	53.64***	2.07*	126.76**	0.29	–	–	–	–
E × GCA_m_ /sets	24	0.75	0.95	0.54	0.33	1.59	120.18	0.18	–	–	–	–
E × GCA_f_ /sets	24	4.04**	0.90	1.36*	0.37	2.17	191.64***	0.38	–	–	–	–
E × SCA/sets	96	2.54	0.88	0.84	0.28	1.85	54.00	0.25	–	–	–	–
Pooled Error	288	2.08	0.98	0.85	0.25	1.49	68.12	0.27	–	–	–	–

*, **, *** Significant at the 0.05, 0.01, and 0.001 probability levels, respectively. BHC, Bad husk cover; EASP, Ear aspect; ERC, Ear circumference; ERL, Ear length; EROT, Percentage of rotten ears; HSL, Husk length; HSN, Husk number; HSW, Husk width; PASP, Plant aspect; TBL, Tassel branch length; TBN, Tassel branch number.

The G × E interaction was significant (*P*< 0.01) for all traits under artificial *Striga* infestation except for EROT (**Table 1**). However, the G × E interaction was not significant for husk traits (HSL, HSW and BHC) and EROT under rainfed conditions, and ear aspect under managed drought stress conditions (**Table 2**). The GCA_m_/sets × E interaction was significant (*P*< 0.05) for HSN, BHC, and TBL under artificial *Striga* infestation, while GCA_f_/sets × E interaction was significant (*P*< 0.05) for ear length (ERL), BHC, TBL, and plant aspect (PASP). In contrast, both GCA_m_/sets × E and GCA_f_/sets × E interactions were significant for HSL, ear circumference (ERC), and tassel branch number (TBN) under rainfed conditions.

**Figure 1:**
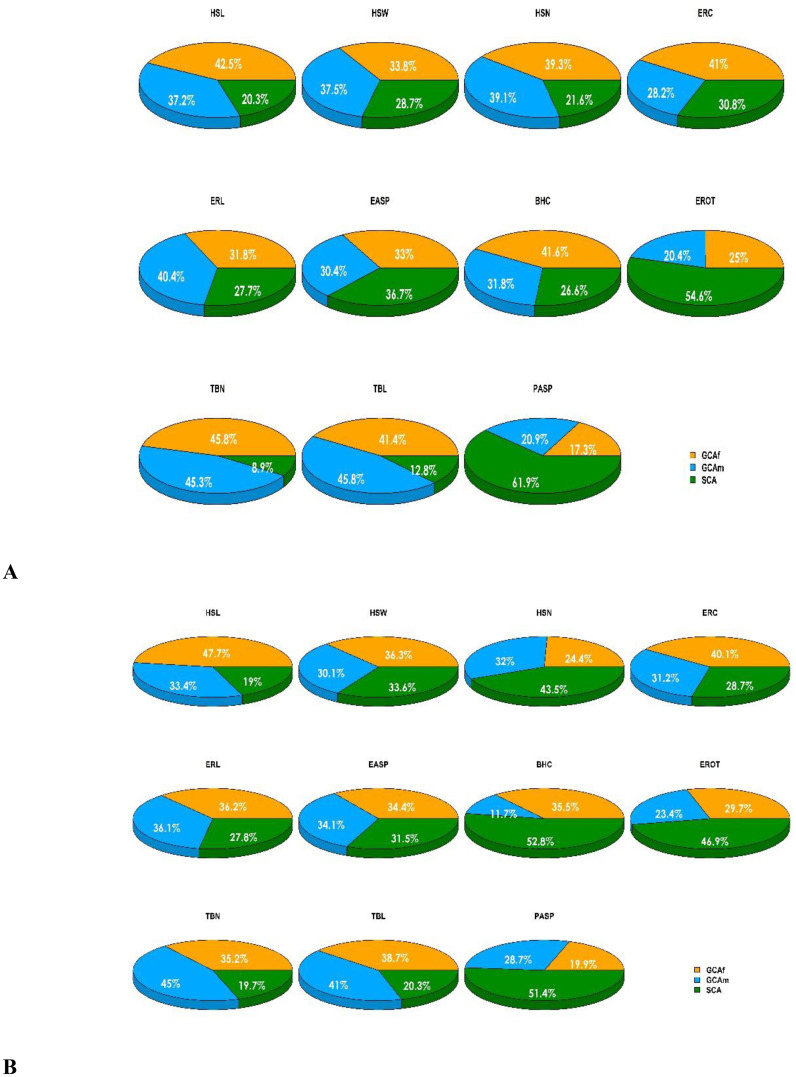
Graphical representation of the sums of squares contribution for husk, ear, tassel, and plant aspect traits under two conditions: **(A)** artificial *Striga* infestation, and **(B)** rainfed conditions. BHC, Bad husk cover; EASP, Ear aspect; ERL, Ear length; ERC, Ear circumference; EROT, Percentage of rotten ears; HSL, Husk length; HSN, Husk number; HSW, Husk width; PASP, Plant aspect; TBL, Tassel branch length; TBN, Tassel branch number.

The partitioning of the genotype sums of squares into GCA (GCA_f_ + GCA_m_) and SCA revealed that GCA accounted for 71.3 to 79.7% of the total variation for husk traits (HSL, HSW, HSN) and 69.2 to 72.2% for ear traits (ERC, ERL) among hybrids under artificial *Striga* infestation (**Figure 1**). Similarly, GCA accounted for a larger proportion of the variation among hybrids for tassel traits TBN and TBL (87.2 to 91.1%) and BHC (73.4%). Under rainfed conditions, GCA accounted for a larger proportion to the total variation among hybrids, contributing 56.4% to 81.1% for husk traits, 71.3% to 72.3% for ear traits, and 79.7% to 82.2% for tassel traits, while SCA sums of squares explained 52.8% of the variation among hybrids. Under managed drought stress conditions, GCA sums of squares were of greater magnitude than SCA sum of squares for all measured traits (**Supplementary Table S3**). A comparison of the contributions of GCA_m_ and GCA_f_ showed that GCA_f_ was greater than GCA_m_ for HSL, ERC, and BHC, while both effects were equal in magnitude for HSN and TBN under artificial *Striga* infestation. Under rainfed conditions GCA_f_ was greater than GCA_m_ for HSL, HSW, ERC, BHC, and EROT, but both effects were equal in magnitude for ERL and ear aspect (EASP).

### Means, variance components, and broad-sense heritability

**Figure 2:**
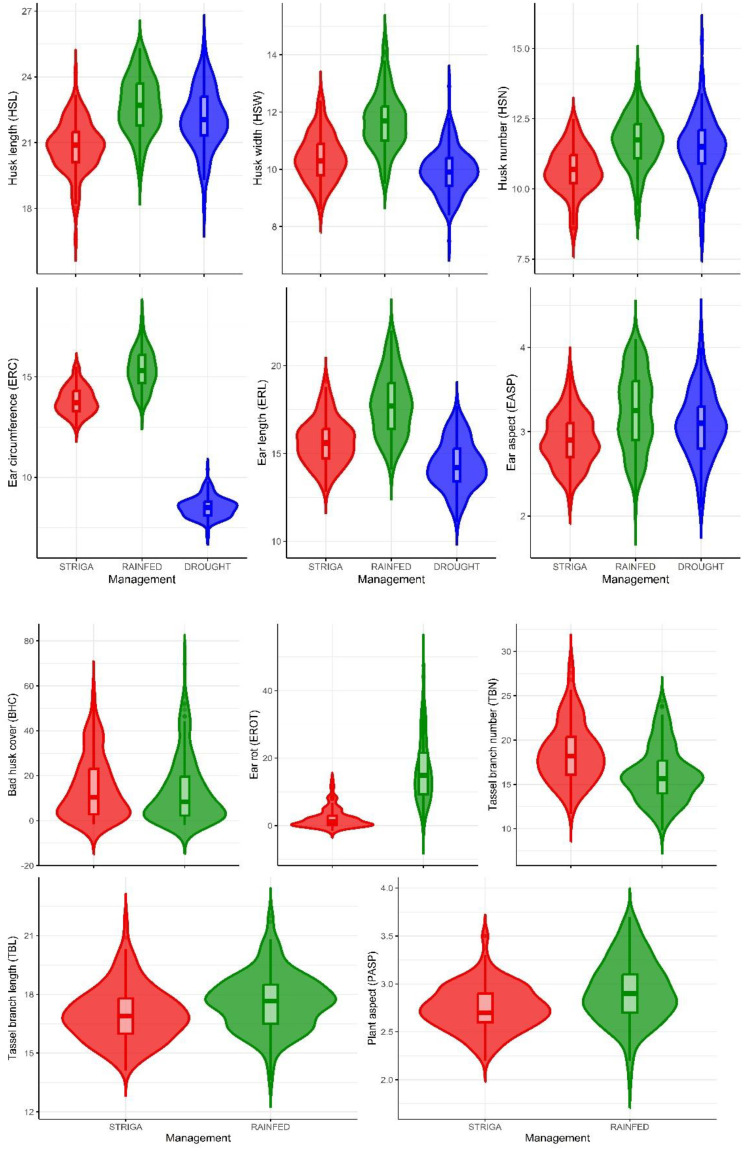
Means of husk, ear, tassel, and plant aspect traits of 150 maize hybrids. Red, green, and blue violin plots represent artificial *Striga* infestation, rainfed, and managed drought stress conditions, respectively.

The summary statistics for traits measured under contrasting management conditions are shown in **Table 3**; **Figure 2**. The mean HSL showed slight variation across the three management conditions, with values of 20.8 cm under artificial *Striga* infestation, 22.7 cm under rainfed conditions, and 22.1 cm under managed drought stress conditions. The means for HSN were similar for rainfed conditions and managed drought stress, but slightly lower for artificial *Striga* infestation. The ear traits ERC and ERL showed smaller values under stressed conditions, with means of 13.8 cm and 15.7 cm under artificial *Striga* infestation, and 8.5 cm and 14.3 cm under managed drought stress. In contrast, under rainfed conditions, the means were higher: 15.4 cm for ERC and 17.8 cm for ERL. The means for tassel traits TBL and TBN were largely identical under artificial *Striga* infestation and rainfed conditions.

**Table 3:** Summary statistics, variance component and broad-sense heritability (*H* ^2^) estimates for husk, ear, and tassel traits of 150 maize hybrids under artificial *Striga* infestation, rainfed, and managed drought stress conditions.

Trait	Unit	Mean	Range	LSD_0.05_	$\sigma_{G}^{2}$	$\sigma_{E}^{2}$	$\sigma_{GE}^{2}$	$\sigma_{\epsilon }^{2}$	*H^2^*
Artificial *Striga* infestation									
HSL^a^	cm	20.8	17.1 – 23.7	1.06	1.13***	1.00	0.09	1.77	0.89
HSW	cm	10.4	8.8 – 12.1	0.86	0.50***	0.29	0.02	1.40	0.85
HSN	count	10.7	8.7 – 12.2	0.78	0.55***	0.63	0.09*	0.97	0.89
ERC	cm	13.8	12.6 – 15.3	0.56	0.26***	0.60	0.03	0.51	0.92
ERL	cm	15.7	12.8 – 18.8	1.06	0.79***	1.36	0.07	2.00	0.93
BHC	%	14.6	3.9 – 43.1	18.01	54.48***	269.36	91.95***	109.69	0.67
EROT	%	2.2	2.0 – 2.5	1.45	0.33	0.20	1.10	18.07	0.04
EASP	1–5	2.9	2.4 – 3.52	0.37	0.05***	0.01	0.08	0.19	0.79
TBL	cm	16.9	14.4 – 21.3	1.22	1.46***	0.09	0.21***	1.10	0.90
TBN	count	18.6	12.1 – 28.2	2.17	10.38***	0.49	0.04	5.60	0.95
PASP	1–5	2.7	2.6 – 3.1	0.32	0.01*	0.01	0.03	0.12	0.41
Rainfed conditions									
HSL	cm	22.7	20.2 – 24.7	1.51	1.11***	0.00	0.09	1.26	0.79
HSW	cm	11.7	10.3 – 13.6	1.30	0.54***	0.00	0.00	1.64	0.73
HSN	count	11.7	9.9 – 13.5	1.19	0.68***	0.79	0.03	1.64	0.76
ERC	cm	15.4	13.7 – 17.5	1.02	0.29***	0.24	0.12**	0.37	0.84
ERL	cm	17.8	14. 5 – 21.6	1.55	1.04***	0.95	0.20**	0.84	0.90
BHC	%	13.3	3.1 – 50.5	19.31	77.39***	26.61	0.00	292.85	0.67
EROT	%	16.5	7.9 – 31.7	12.87	36.32***	130.75	8.36	137.74	0.58
EASP	1–5	3.3	2.4 – 3.9	0.56	0.07***	0.02	0.02*	0.12	0.74
TBL	cm	17.7	14.6 – 20.8	1.75	1.39***	0.50	0.00	2.45	0.78
TBN	count	16.1	11.3 – 22.6	3.48	7.29***	7.38	1.10*	6.92	0.82
PASP	1–5	2.9	2.4 – 3.4	0.45	0.03**	0.00	0.02	0.16	0.57
Managed drought stress									
HSL	cm	22.1	19.4 – 24.4	1.78	1.04***	0.14	0.17	2.10	0.67
HSW	cm	9.9	8.6 – 11.0	1.03	0.30***	0.50	0.00	0.95	0.59
HSN	count	11.5	8.9 – 14.5	1.22	0.89	0.12	0.02	0.83	0.80
ERC	cm	8.5	7.54 – 9.8	0.67	0.14***	26.65	0.03	0.25	0.71
ERL	cm	14.3	11.9 – 16.8	1.69	0.89***	0.04	0.19	1.49	0.75
BHC	%	4.2	2.0 – 16.2	7.56	10.17	0.00	26.05**	64.19	0.42
EASP	1–5	3.1	2.5 – 3.7	0.55	0.07***	0.01	0.00	0.26	0.62

*, **, *** Significant at the 0.05, 0.01, and 0.001 probability levels, respectively.

a BHC, Bad husk cover; EASP, Ear aspect; ERL, Ear length; ERC, Ear circumference; EROT, Percentage of rotten ears; HSL, Husk length; HSN, Husk number; HSW, Husk width; PASP, Plant aspect; TBL, Tassel branch length; TBN, Tassel branch number. $\sigma_{G}^{2}$ , $\sigma_{E}^{2}$ , $\sigma_{GE}^{2}$ and $\sigma_{\epsilon }^{2}$ refer to genetic, environment, genotype × environment, and residual variance, respectively.

Genetic variance estimates were significantly different from zero for all traits under all management conditions, except for EROT under artificial *Striga* infestation and HSN and BHC under managed drought stress. Under artificial *Striga* infestation, genetic variance exceeded environmental variance for two husk traits (HSL and HSW), EASP, and tassel traits (TBL and TBN). Broad-sense heritability estimates were generally high across all management conditions, ranging from 0.67 to 0.95 (**Table 3**). Under artificial *Striga* infestation, husk traits (0.85 to 0.89), ear traits (0.92 to 0.93), and tassel traits (0.90 to 0.95) exhibited particularly high heritability estimates. Exceptions included moderate heritability for PASP, EROT, HSW, and BHC (0.41 to 0.59) and low heritability for EROT under artificial *Striga* infestation.

### Combining ability effects

The GCA effects for husk, ear, and tassel traits under artificial *Striga* infestation are shown in **Table 4**. Nine inbred lines including TZSTR189, CKDHL171092, CKDHL171119, CKDHL171527, CKL17604, CKL17517, CKL17531, CKL17719, and CML610A exhibited significant (*P*< 0.05) positive GCA_f_ and GCA_m_ effects for HSL. For HSW, significant positive GCA_f_ and GCA_m_ effects were observed in eight inbred lines, with two of these lines displaying significant positive GCA_f_ and GCA_m_ effects for both HSL and HSW. For HSN, one line (CKDHL171162) had significant negative GCA_f_ and GCA_m_ effects, while five lines showed significant negative GCA_f_ effects, and 11 lines showed significant negative GCA_m_ effects. Conversely, three lines (CKL17513, CML543 and CML312) showed significant positive GCA_f_ effects and significant negative GCA_m_ effects for HSN. The GCA effects for ERC and ERL varied among the lines. Seven inbred lines exhibited significant (*P*< 0.05) positive GCA_f_ and GCA_m_ effects for ERC, while eight lines showed significant positive GCA_f_ and GCA_m_ effects for ERL. Notably, CKL17571 showed significant positive GCA_f_ and GCA_m_ effects for both ERC and ERL. For tassel traits, 11 inbred lines exhibited significant negative GCA_f_ and GCA_m_ effects for TBL, while 13 inbred lines showed significant negative GCA_f_ and GCA_m_ effects for TBN. Four lines (TEISTR1159, CKL17961, CKDHL171527 and CKL17611) showed significant negative GCA_f_ and GCA_m_ effects for both TBL and TBN.

**Table 4:** Estimates of general combining ability effects of females (GCA_f_) and males (GCA_m_) for husk, ear, and tassel traits of 30 tropical maize inbred lines evaluated under artificial *Striga* infestation across five environments in 2020 and 2021.

Name	HSL^a^		HSW		HSN		ERC		ERL		TBL		TBN	
	GCA_f_	GCA_m_	GCA_f_	GCA_m_	GCA_f_	GCA_m_	GCA_f_	GCA_m_	GCA_f_	GCA_m_	GCA_f_	GCA_m_	GCA_f_	GCA_m_
TEISTR1156	-0.77***	-0.45*	-0.44*	-0.20	0.03	0.00	-0.16	0.03	0.50*	0.71**	0.00	0.08	2.61***	0.86*
TEISTR1157	0.29	0.02	-0.08	0.01	0.30*	-0.11	-0.06	-0.46***	0.28	0.09	-0.11	-0.41*	0.14	1.18**
TEISTR1158	-0.55*	-0.36	-0.10	-0.23	-0.03	0.19	-0.15	0.15	-0.45*	-0.32	0.19	0.46**	1.62***	2.13***
TEISTR1159	0.09	-0.33	-0.11	-0.15	-0.14	-0.31*	0.12	0.14	-0.35	-0.48*	-0.31*	-1.06***	-1.45***	-1.00**
TZSTR189	0.94***	1.12***	0.73***	0.57**	-0.16	0.24	0.25*	0.13	0.02	0.01	0.24	0.93***	-2.92***	-3.17***
CKDHL17888	-0.34	-0.09	-0.10	-0.12	-0.82***	0.51**	-0.40**	-0.39**	0.21	0.57*	0.51**	0.68***	1.96***	1.54***
CKDHL17961	-0.90***	-0.94***	0.75***	0.61**	-0.08	-0.83***	0.07	0.24*	-0.25	-0.31	-0.83***	-0.67***	-0.73*	-1.04**
CKDHL171092	1.03***	0.69**	0.23	0.56**	0.63***	0.18	0.55***	0.34**	-0.68**	-0.61**	0.18	-0.43*	-1.84***	-1.90***
CKDHL171119	0.52*	0.82***	-0.71***	-0.57**	0.79***	0.58**	0.06	0.14	0.94***	0.75**	0.58**	0.54**	-1.92***	-0.86*
CKDHL171162	-0.31	-0.48*	-0.17	-0.48*	-0.52**	-0.44**	-0.28*	-0.33**	-0.22	-0.39	-0.44**	-0.13	2.54***	2.25***
CKDHL171267	-1.05***	-1.11***	0.24	0.10	0.01	-1.15***	-0.13	0.07	0.44*	0.78**	-1.15***	-0.58**	5.79***	5.23***
CKDHL171357	0.35	0.04	-0.06	-0.04	0.15	0.32*	-0.19	-0.22*	0.60**	0.66**	0.32*	0.09	2.38***	2.33***
CKDHL171514	0.20	0.72**	-0.44*	-0.52**	-0.45**	0.38*	0.24*	-0.02	0.18	-0.09	0.38*	0.48**	-2.89***	-3.10***
CKDHL171520	0.11	-0.19	-0.10	-0.08	-0.04	1.54***	-0.20*	-0.29*	-0.52*	-0.76**	1.54***	0.89***	-3.33***	-3.57***
CKDHL171527	0.39*	0.54*	0.36*	0.54**	0.34*	-1.09***	0.27*	0.46***	-0.70**	-0.59*	-1.09***	-0.88***	-1.95***	-0.89*
CKDHL171564	-0.68**	-0.60**	-0.70***	-0.96***	0.32*	-1.34***	0.00	0.14	-0.73**	-0.40*	-1.34***	-1.09***	2.93***	3.90***
CKL17535	0.15	0.19	0.50**	0.73***	-0.42***	1.59***	-0.23*	-0.29*	1.15***	1.02***	1.59***	1.58***	-1.37***	-2.20***
CKL17571	0.17	0.34	1.15***	0.92***	0.23	1.05***	0.36**	0.64***	0.54*	0.86**	1.05***	0.83***	-0.31	-0.76*
CKL17604	0.81***	0.45*	-0.58**	-0.55**	0.05	-0.76***	-0.21*	-0.16	-0.25	-0.75**	-0.76***	-0.83***	-0.11	0.97*
CKL17611	-0.45*	-0.38*	-0.38*	-0.14	-0.18	-0.53**	0.09	-0.34**	-0.70**	-0.72**	-0.53**	-0.50**	-1.14**	-1.91***
CKL17508	0.40*	-0.12	-0.64**	-0.38*	-0.32*	0.28*	0.33**	0.39**	-0.05	-0.33	0.28	0.70***	-3.76***	-3.83***
CKL17513	-1.64***	-2.25***	0.53**	0.38*	0.79***	-0.35*	0.42***	0.54***	-0.71**	-0.53*	-0.35*	-1.18***	0.95*	0.81*
CKL17517	0.39*	1.08***	-0.45*	-0.50**	-0.23	-0.80***	-0.07	-0.03	0.12	0.09	-0.80***	-0.85***	2.92***	1.63***
CKL17531	0.75**	0.86***	0.24	0.11	-0.19	-0.01	-0.08	-0.32**	0.40*	-0.06	-0.01	-0.29	1.14**	2.55***
CKL17650	0.10	0.44	0.33*	0.40*	-0.05	0.89***	-0.61***	-0.58***	0.24	0.82**	0.89***	1.63***	-1.25**	-1.16**
CKL17719	0.96***	0.82***	-0.46*	-0.62**	-1.56***	1.27***	-0.71***	-0.69***	1.20***	0.43*	1.27***	0.67***	1.30**	2.07***
CML610A	0.78***	0.51*	0.20	0.40*	0.01	0.09	-0.05	-0.44***	1.02***	0.45*	0.09	-0.21	-1.90***	-1.63***
CKL12128	-1.33***	-1.09***	0.40*	0.05	-0.02	1.18***	0.22*	0.43***	-0.21	-0.12	1.18***	1.07***	0.69*	0.75*
CML543	0.11	-0.28	0.51**	0.34*	0.51**	-1.89***	0.44***	0.49***	-1.33***	-0.57*	-1.89***	-1.14***	-0.07	0.01
CML312	-0.51*	0.03	-0.65***	-0.18	1.06***	-0.65***	0.10	0.21*	-0.68**	-0.19	-0.65***	-0.40*	-0.03	-1.19**
SE	0.19		0.17		0.14		0.10		0.20		0.15		0.33	

*, **, *** Significant at the 0.05, 0.01, and 0.001 probability levels, respectively.

a ERC, Ear circumference; ERL, Ear length; HSL, Husk length; HSN, Husk number; HSW, Husk width; TBL, Tassel branch length; TBN, Tassel branch number.

Five inbred lines exhibited significant (*P*< 0.05) desirable (negative) GCA_f_ and GCA_m_ effects for BHC, while four additional lines showed significant desirable GCA_f_ effects (**Supplementary Table S4**). Furthermore, two lines (CKDHL171092 and CKL17508) showed significant desirable GCA_m_ effects for BHC. Inbred lines CKDHL171514 and CKL17650 showed significant desirable (negative) GCA_m_ effects for EROT. Favorable (negative) significant GCA_f_ and GCA_m_ effects for EASP were observed on five lines, while two lines showed favorable GCA_m_ effects.

The GCA effects for husk, ear, and tassel traits under rainfed conditions are presented in **Table 5**. Seven inbred lines showed significant (*P*< 0.05) positive GCA_f_ and GCA_m_ effects for HSL. Additionally, two lines (CKL17650 and CML543) had significant positive GCA_m_ effects. Only two lines exhibited significant positive GCA_f_ and GCA_m_ effects for HSW. However, six additional lines showed either significant positive GCA_f_ or GCA_m_ effects. For HSN, three lines exhibited significant negative GCA_f_ and GCA_m_ effects for HSN, with three more lines showing either significant negative GCA_f_ or GCA_m_ effects. Significant positive GCA_f_ and GCA_m_ effects for ERC were observed in five lines (TZSTR189, CKDHL171092, CKL17571, CKL17513, and CML543), while six lines (CKDHL171119, CKDHL171267, CKL17535, CKL17571, CKL17531, and CML610A) showed significant positive GCA_f_ and GCA_m_ effects for ERL. An additional five lines showed significant positive GCA_m_ effects. Four lines (CKDHL171527, CKL17513, CKL17517, and CML543) showed significant GCA_f_ and GCA_m_ effects for reduced TBL, and seven more lines had either significant GCA_f_ or GCA_m_ effects. In contrast, seven lines showed significant GCA_f_ and GCA_m_ effects for reduced TBN. Additionally, three lines exhibited significant GCA_f_ effects, and two lines showed significant GCA_m_ effects for reduced TBN.

**Table 5:** Estimates of general combining ability effects of females (GCA_f_) and males (GCA_m_) for husk, ear, and tassel traits of 30 tropical maize inbred lines evaluated under rainfed conditions in 2020 and 2021.

Name	HSL^a^		HSW		HSN		ERC		ERL		TBL		TBN	
	GCA_f_	GCA_m_	GCA_f_	GCA_m_	GCA_f_	GCA_m_	GCA_f_	GCA_m_	GCA_f_	GCA_m_	GCA_f_	GCA_m_	GCA_f_	GCA_m_
TEISTR1156	-0.12	-0.10	-0.22	0.07	0.47	-0.28	-0.14	-0.35*	0.29	0.42*	0.43	0.71*	1.43*	0.27
TEISTR1157	0.16	0.08	-0.31	-0.64*	-0.22	0.30	0.12	-0.26	0.39	0.64**	-0.20	0.18	0.96	0.54
TEISTR1158	-0.02	-0.56*	0.18	-0.10	0.24	-0.06	-0.26	-0.16	-0.20	-0.74**	0.46	0.50	1.45*	2.03**
TEISTR1159	-0.55*	-0.72*	0.08	-0.11	-0.31	-0.01	-0.01	0.19	-0.74**	-0.45*	-0.21	-0.89*	-1.90**	-1.03
TZSTR189	0.53*	1.29***	0.27	0.77*	-0.18	0.05	0.28*	0.59***	0.26	0.13	-0.48	-0.48	-1.94**	-1.81**
CKDHL17888	-0.68*	-0.76*	-0.36	-0.42	-0.68*	-0.65*	-0.55***	-0.68***	-0.05	0.27	0.19	0.63	1.68*	1.31*
CKDHL17961	-0.74*	-0.93**	0.44	0.10	-0.11	0.26	-0.13	0.01	-0.52*	-0.74**	-0.38	-0.71*	-0.60	-1.50*
CKDHL171092	0.88**	0.97**	0.31	0.90**	0.65*	0.29	0.75***	0.72***	-0.68**	-0.57*	-0.55	-0.34	-0.80	-0.14
CKDHL171119	0.66*	1.25***	-0.08	-0.19	0.46	0.37	0.02	-0.18	1.25***	0.83***	1.68***	0.45	-2.90***	-0.99
CKDHL171162	-0.11	-0.52*	-0.31	-0.39	-0.33	-0.27	-0.09	0.12	0.00	0.21	-0.94*	-0.03	2.61***	1.31*
CKDHL171267	0.11	-0.62*	0.56	-0.17	0.56	0.45	0.47**	0.22	1.16***	0.75**	-0.66	-0.68	4.65***	4.12***
CKDHL171357	0.01	0.05	-0.15	-0.19	-0.18	0.48	-0.63***	-0.32*	0.21	0.49*	0.38	0.41	1.77**	1.39*
CKDHL171514	0.09	0.31	-0.53	-0.38	-0.72*	-0.48	-0.04	-0.32*	0.00	-0.19	0.54	0.72*	-2.38***	-2.91***
CKDHL171520	-0.78**	-0.26	-0.13	0.26	-0.20	-0.76*	-0.05	-0.13	-0.43*	-0.56*	0.93*	0.41	-2.42***	-2.42***
CKDHL171527	0.57*	0.51*	0.25	0.48	0.54	0.31	0.25	0.54**	-0.94***	-0.49*	-1.19**	-0.87*	-1.62*	-0.17
CKDHL171564	-0.78**	-0.36	-0.88*	-0.21	-0.32	0.22	-0.08	0.01	-0.28	-0.32	-1.26**	-0.59	2.22**	1.75**
CKL17535	0.34	0.10	0.49	0.55	-1.08**	-0.73*	-0.08	-0.16	0.84**	1.21***	2.09***	1.42***	-2.32**	-1.21*
CKL17571	0.66*	0.67*	0.93**	1.10**	1.38***	0.20	0.54**	0.83***	1.07***	1.08***	0.47	0.29	-0.34	-0.63
CKL17604	0.24	0.02	-0.32	-0.45	0.10	0.30	-0.49**	-0.07	-0.48*	-0.42*	0.00	-0.91*	0.56	1.91**
CKL17611	-0.46	-0.43	-0.21	-0.99**	-0.08	0.01	0.11	-0.61***	-1.16***	-1.54***	-1.29**	-0.21	-0.11	-1.81**
CKL17508	-0.26	0.00	-1.18***	-0.35	-0.45	-0.70*	0.02	0.27*	-0.32	-0.44*	0.68	1.00*	-3.36***	-3.92***
CKL17513	-1.73***	-2.20***	1.08**	-0.21	0.41	0.49	0.62***	0.50**	-0.10	-0.15	-0.76*	-1.03*	-0.74	1.90**
CKL17517	0.32	-0.28	-0.34	-0.94**	-0.38	-0.14	0.06	-0.15	-0.24	-0.24	-1.30**	-1.57***	3.95***	1.15
CKL17531	1.54***	1.41***	0.16	0.64*	0.13	0.02	-0.12	-0.27*	1.14***	0.99***	-0.12	-0.68	2.56***	2.81***
CKL17650	0.13	1.07***	0.27	0.86*	0.30	0.34	-0.57***	-0.35*	-0.48*	-0.17	1.50***	2.28***	-2.40***	-1.94**
CKL17719	0.26	-0.07	-0.92**	-1.06**	-1.57***	-1.58***	-0.68***	-0.76***	-0.15	0.62**	1.07**	1.01*	0.52	0.90
CML610A	1.17***	1.16***	0.66*	0.17	0.37	0.09	-0.04	-0.03	1.56***	0.63**	-0.22	0.45	-1.44*	-1.81**
CKL12128	-1.16***	-1.41***	0.13	-0.06	-0.13	-0.20	-0.01	-0.18	-0.90***	-1.44***	0.74*	0.64	0.91	0.47
CML543	0.41	0.67*	0.64*	1.08**	0.68*	0.88**	0.70***	0.81***	0.10	0.54*	-0.92*	-0.77*	-0.23	0.47
CML312	-0.68*	-0.35	-0.50	-0.14	0.66*	0.81*	0.04	0.16	-0.62**	-0.35	-0.67	-1.34**	0.24	-0.03
SE	0.26		0.30		0.29		0.14		0.21		0.36		0.59	

*, **, *** Significant at the 0.05, 0.01, and 0.001 probability levels, respectively.

a ERC, Ear circumference; ERL, Ear length; HSL, Husk length; HSN, Husk number; HSW, Husk width; TBL, Tassel branch length; TBN, Tassel branch number.

The GCA effects for agronomic traits under rainfed conditions are shown in **Supplementary Table S4**. One inbred line exhibited significant (*P*< 0.05) desirable (negative) GCA_f_ and GCA_m_ effects for BHC, while three additional lines showed significant desirable GCA_m_ effects. Inbred lines TEISTR1157 and CKDHL171527 showed significant desirable (negative) GCA_f_ and GCA_m_ effects for EROT, with five other lines exhibiting significant desirable GCA_f_ or GCA_m_ effects. Five inbred lines showed desirable significant (*P*< 0.05) GCA_f_ and GCA_m_ effects for EASP. Four lines showed significant negative GCA_f_ effects for PASP, while two lines exhibited significant negative GCA_m_ effects.

Under managed drought stress conditions, six inbred lines showed significant (*P*< 0.05) positive GCA_f_ effects for HSL, while five lines exhibited significant positive GCA_m_ effects (**Table 6**). Only one line (CML610A) showed significant positive GCA_f_ and GCA_m_ effects for HSL. Similarly, six inbred lines showed significant positive GCA_f_ effects for HSW, with two lines (CKL17535 and CML610A) showing significant positive GCA_f_ and GCA_m_ effects. Seven lines exhibited significant negative GCA_f_ and GCA_m_ effects for HSN, while five lines showed significant positive GCA_f_ and GCA_m_ effects for the same trait. Significant and positive GCA_f_ and GCA_m_ effects for ERC were exhibited by five lines (TEISTR1159, CKL17571, CKL17513, CKL12128, and CML543), while four lines (CKDHL171267, CKDHL171357, CKL17535, and CKL17531) exhibited significant positive GCA_f_ and GCA_m_ effects for ERL. Six inbred lines showed significant negative GCA_f_ effects for TBL, and three lines exhibited significant negative GCA_m_ effects. Two lines showed significant negative GCA_f_ and GCA_m_ effects, with an additional five lines exhibiting significant negative GCA_f_ effects.

**Table 6:** Estimates of general combining ability effects of females (GCA_f_) and males (GCA_m_) for husk, ear, and tassel traits of 30 lines evaluated under managed drought stress conditions at Kiboko in 2020 and 2021.

Name	HSL^a^		HSW		HSN		ERC		ERL		TBL		TBN	
	GCA_f_	GCA_m_	GCA_f_	GCA_m_	GCA_f_	GCA_m_	GCA_f_	GCA_m_	GCA_f_	GCA_m_	GCA_f_	GCA_m_	GCA_f_	GCA_m_
TEISTR1156	-0.58	-0.18	-0.27	-0.17	0.02	0.44*	-0.11	-0.03	0.28	0.65*	-0.76	-0.66	-0.05	-0.38
TEISTR1157	0.43	0.16	-0.23	-0.24	0.42*	0.01	-0.09	-0.17	0.18	0.08	-0.46	-0.44	1.04	0.10
TEISTR1158	-0.48	-0.67*	0.09	0.03	-0.04	0.14	-0.12	-0.04	-0.46	-0.62*	-0.22	0.39	1.22*	0.72
TEISTR1159	-0.09	0.32	0.09	-0.05	-0.04	0.05	0.27*	0.22*	-0.26	-0.28	0.92*	-0.05	-1.07	0.56
TZSTR189	0.72*	0.37	0.33	0.41	-0.36	-0.64**	0.06	0.03	0.27	0.16	0.53	0.76	-1.14	-1.00
CKDHL17888	0.67*	0.46	0.10	-0.09	-0.58*	-0.85***	-0.19	-0.18	0.39	0.51	1.25**	0.36	2.14**	2.86***
CKDHL17961	-1.05**	-0.91*	-0.03	0.06	-0.34	-0.26	-0.11	0.13	-0.57*	0.10	-0.96*	-0.51	-1.87**	-0.99
CKDHL171092	1.12**	0.35	0.50*	0.12	0.62**	0.32	0.43**	-0.07	0.06	-1.26***	-0.46	-0.48	-0.30	-2.04**
CKDHL171119	0.34	0.51	-0.16	-0.10	0.58*	0.61**	-0.02	0.01	0.53*	0.38	0.06	0.64	-0.22	-0.77
CKDHL171162	-1.07**	-0.42	-0.41	0.01	-0.29	0.18	-0.11	0.10	-0.42	0.28	0.11	-0.01	0.25	0.95
CKDHL171267	-0.29	-0.60	0.60*	0.08	0.14	0.03	0.01	-0.07	0.72*	0.70*	-1.03*	0.55	4.21***	1.93**
CKDHL171357	0.12	-0.25	-0.13	0.16	0.45*	0.26	-0.01	-0.15	0.77*	0.66*	0.69	-0.87*	1.58*	-0.11
CKDHL171514	-0.05	0.65*	-0.71**	-0.64**	-0.45*	-0.52*	0.02	-0.14	0.17	0.07	-0.12	0.77	-1.44*	-0.90
CKDHL171520	-0.14	-0.21	0.22	-0.18	-0.09	-0.06	-0.06	0.07	-0.81*	-0.83**	1.93***	0.24	-2.04**	-0.18
CKDHL171527	0.36	0.41	0.03	0.58*	-0.05	0.30	0.04	0.29*	-0.85**	-0.59*	-1.47**	-0.68	-2.31**	-0.74
CKDHL171564	-1.00**	-0.07	-0.49*	-0.53*	0.96***	0.80**	0.07	0.12	-0.43	0.31	0.42	-0.83*	2.37**	1.80**
CKL17535	-0.30	-0.24	0.43*	0.50*	-1.01***	-0.64**	-0.09	-0.05	1.54***	1.35***	0.43	0.99*	-1.69*	-0.02
CKL17571	0.70*	0.28	0.34	0.87***	0.81**	0.49*	0.33**	0.50***	0.34	0.27	0.11	0.26	0.35	-0.03
CKL17604	0.63	0.78*	-0.34	-0.55*	-0.02	0.51*	-0.18	-0.03	-0.59*	-0.54*	-1.72***	0.09	0.46	-0.25
CKL17611	-0.03	-0.75*	0.05	-0.30	-0.74**	-1.16***	-0.13	-0.54***	-0.86**	-1.39***	0.77	-0.51	-1.49*	-1.50*
CKL17508	0.46	0.24	-0.44*	-0.33	-0.93***	-0.66**	-0.11	0.16	-0.80*	-1.02**	0.46	0.02	-1.42*	-2.00**
CKL17513	-1.01**	-2.10***	0.54*	0.36	1.51***	1.06***	0.47***	0.29*	-0.22	-0.41	-1.16*	-0.08	2.49***	2.18**
CKL17517	0.06	-0.53	-0.59*	-0.68**	-0.45*	-0.61*	-0.10	-0.18	-0.05	-0.26	0.74	0.25	-1.08	-0.19
CKL17531	0.35	0.86*	0.37	0.40	-0.12	0.09	-0.16	-0.09	0.71*	0.56*	-0.08	-0.79	-0.25	-0.28
CKL17650	0.14	1.53***	0.12	0.25	-0.01	0.12	-0.09	-0.18	0.36	1.13***	0.05	0.60	0.26	0.29
CKL17719	-0.41	-0.27	-0.82**	-0.32	-1.47***	-1.60***	-0.85***	-0.51***	0.25	-0.09	1.18*	0.44	-0.97	0.40
CML610A	0.73*	1.22**	0.43*	0.55*	-0.16	-0.27	-0.16	-0.25*	0.73*	0.48	0.12	-0.14	-0.34	0.55
CKL12128	-0.83*	-1.31***	0.26	-0.29	-0.14	-0.11	0.41**	0.42**	0.25	0.03	0.42	-0.05	0.31	0.15
CML543	0.91*	0.08	0.78**	0.35	0.36	0.52*	0.50***	0.36**	-0.46	-0.17	-0.70	-0.85*	0.13	0.50
CML312	-0.40	0.27	-0.65**	-0.29	1.41***	1.47***	0.10	-0.02	-0.77*	-0.24	-1.01*	0.59	0.88	-1.59*
SE	0.33		0.22		0.21		0.11		0.27		0.40		0.61	

*, **, *** Significant at the 0.05, 0.01, and 0.001 probability levels, respectively.

a ERC, Ear circumference; ERL, Ear length; HSL, Husk length; HSN, Husk number; HSW, Husk width; TBL, Tassel branch length; TBN, Tassel branch number.

### ANOVA for inbred lines and *per se* performance

The combined ANOVA for inbred lines revealed significant environment (E), genotype (G), and G × E interaction mean squares for all traits, except for G × E interaction for HSN and BHC under artificial *Striga* infestation (**Supplementary Table S5**). Significant variation attributable to both E and G was observed for the measured traits under rainfed and managed drought stress conditions. Additionally, the G × E interaction was significant for PASP under rainfed conditions, and for BHC and ERC under managed drought stress conditions. Under artificial *Striga* infestation, the mean ERC was 11.69 cm, ranging from 10.82 to 13.03 cm, while ERL ranged from 11.15 to 14.90 cm, with a mean of 12.85 cm. The TBL values ranged from 10.7 to 19.2, and TBN varied from 8 to 24. Under managed drought stress conditions, the average values for ERC and ERL were lower than those recorded under artificial *Striga* infestation. Broad-sense heritability estimates were moderate for BHC under both artificial *Striga* infestation (0.56) and managed drought stress conditions (0.46), as well as for EROT under artificial *Striga* infestation (0.50). For all other traits, except for PASP, broad-sense heritability estimates were high, ranging from 0.67 to 0.95 (**Supplementary Table S5**).

### Genotypic correlations among traits

**Figure 3:**
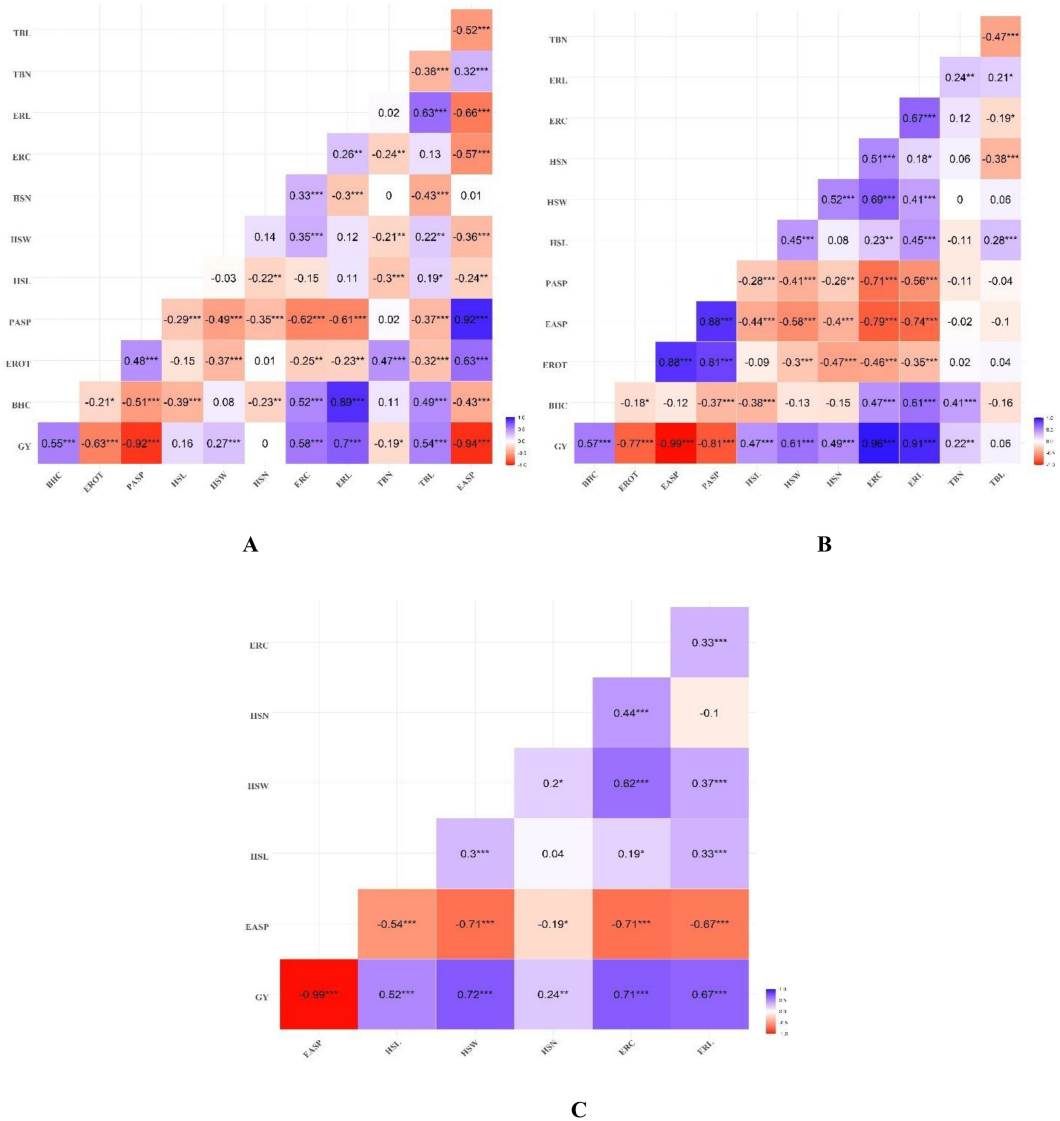
Heatmap of genotypic correlations between grain yield, husk, ear, and tassel related traits under **(A)** artificial *Striga* infestation, **(B)** rainfed, and **(C)** managed drought stress conditions. *, **, *** Significant at 0.05, 0.01, and 0.001 probability levels, respectively.

Genotypic correlations among the measured traits under artificial *Striga* infestation and rainfed conditions are presented in **Figure 3**. Of the 66 trait pairs under artificial *Striga* infestation, 19 exhibited significant positive genotypic correlations, while 31 pairs showed significant negative correlations. Ear-related traits (HSL, HSW, HSN, ERC, ERL) and BHC were negatively correlated with PASP (*r_g_* = –0.29 to –0.61, *P*< 0.001). HSL and HSW had significant (*P*< 0.01) correlations with EASP and TBN. HSW and HSN were positively correlated with ERC (*P*< 0.001). Both ERC and ERL were positively and significantly (*P*< 0.001) correlated with BHC, and ERL was positively correlated with TBL (*P*< 0.001). TBN was negatively correlated with TBL (–0.38, *P*< 0.001). Grain yield (GY) had significant (*P*< 0.001) positive correlations with BHC, HSW, ERC, ERL and TBL. Significant (*P*< 0.05) negative correlations were observed between GY and EROT, PASP, TBN and EASP. Under rainfed conditions, a similar pattern of negative genotypic correlations between PASP and ear related traits (HSL, HSW, HSN, ERC, ERL) and BHC was observed. The husk traits (HSL, HSW, HSN) were positively correlated with ERC and ERL (*r_g_* = 0.18 to 0.69, *P*< 0.05). ERL was also positively correlated with both TBL and TBN (*r_g_* = 0.21 to 0.24, *P*< 0.05). Significant positive correlations (*P*< 0.01) were observed between GY and BHC, husk traits, ERC, ERL, and TBN. Under managed drought stress conditions, genotypic correlations among traits revealed that BHC exhibited a significant negative correlation with all recorded traits (*r_g_* = –0.19 to –0.71, *P*< 0.01) (**Figure 3**). HSL was negatively correlated with both HSW and HSN, while ERC and ERL were positively correlated with both HSW and HSN. Significant (*P*< 0.0) positive correlations between GY and HSL, HSW, HSN, ERC, and ERL was observed.

### Heterosis estimates for ear and tassel traits

**Figure 4:**
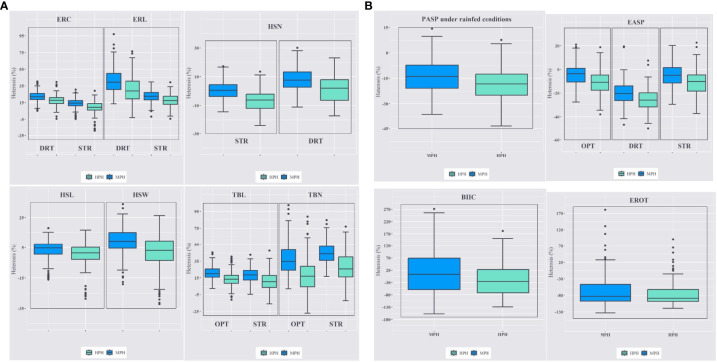
**(A)** Mid-parent and high-parent heterosis estimates for ear, husk and tassel traits under managed drought stress (DRT), artificial *Striga* infestation (STR), and rainfed (OPT) conditions. ERC, ear circumference; ERL, ear length; HSN, husk number; HSL, husk length; HSW, husk width; TBL, tassel branch length; TBN, tassel branch number. **(B)** Mid-parent and high-parent heterosis estimates for plant aspect (PASP), ear aspect (EASP); bad husk cover (BHC), and percentage of rotten ears (EROT) under rainfed, artificial *Striga* infestation, and managed drought stress conditions.

Mid-parent (MPH) and high-parent heterosis (HPH) estimates varied across the three management conditions (**Figure 4**). Under artificial *Striga* infestation, the average MPH for ERL was 22.8%, ranging from -1.57% to 39.90%, with a similar range observed for HPH. Tassel branch number (TBN) exhibited greater heterosis compared to TBL, with MPH of 53.2% and 24%, respectively. Under managed drought stress, MPH and HPH for ERC and ERL were of higher magnitude than those under artificial S*triga* infestation, with a two-fold increase in MPH for ERL. The MPH ranged from 2.27% to 45.50% for TBL and from 1.96% to 102.70% for TBN under rainfed conditions. The range of heterosis for TBL and TBN was larger under rainfed conditions compared to artificial *Striga* infestation.

### Path analysis

**Figure 5:**
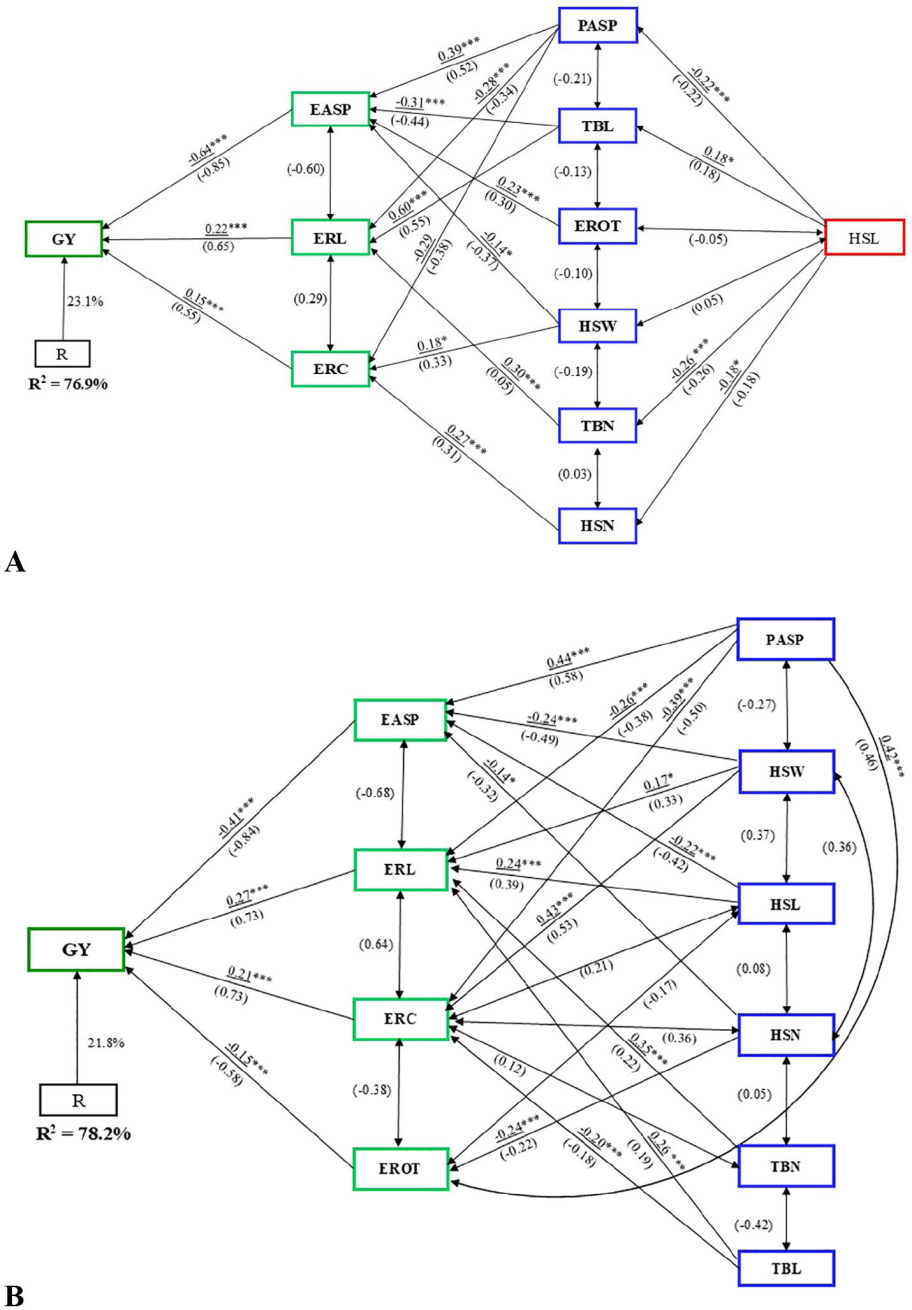
Path analysis model illustrating the causal relationships between grain yield (GY) and husk, ear, tassel, and agronomic traits under **(A)** artificial *Striga* infestation, and **(B)** rainfed conditions. Underlined values represent direct path coefficients, while values in parenthesis indicate correlation coefficients. Single-arrow lines represent path coefficients, while double-arrow lines denote correlation coefficients. EASP, ear aspect; ERC, ear circumference; ERL, ear length; EROT, Percentage of rotten ears; HSL, husk length; HSN, husk number; HSW, husk width; PASP, plant aspect; TBL, tassel branch length; TBN, tassel branch number. *, **, *** Significant at the 0.05 and 0.001 probability levels, respectively.

Path analysis using stepwise regression identified EASP, ERL, and ERC as first-order traits, which together explained 77% of the variation in GY under artificial *Striga* infestation (**Figure 5A**). Ear length and ERC had positive path coefficients (0.22 and 0.15, respectively), while EASP had a negative direct path coefficient of –0.64 with GY. Among the second-order traits, TBL had the largest indirect effect (0.60) on GY. Second-order traits PASP, TBL, and TBN influenced GY indirectly via ERL, while HSW, HSN, and PASP affected GY through ERC.

Under rainfed conditions, path analysis revealed EASP, ERL, ERC, and EROT as first-order traits, accounting for 78% of the variation in GY (**Figure 5B**). ERL and ERC had positive path coefficients of 0.27 and 0.21, respectively, while EASP and EROT had negative direct path coefficients with GY. The rest of the traits were categorized as second-order traits. The second-order traits, except HSN, influenced GY indirectly through ERL. Additionally, PASP, HSW, and TBL had indirect effects on GY via ERC, while HSN and PASP impacted GY through EROT. Among the second-order traits, PASP had the largest indirect effect (0.44), followed by HSW (0.43).

**Figure 6:**
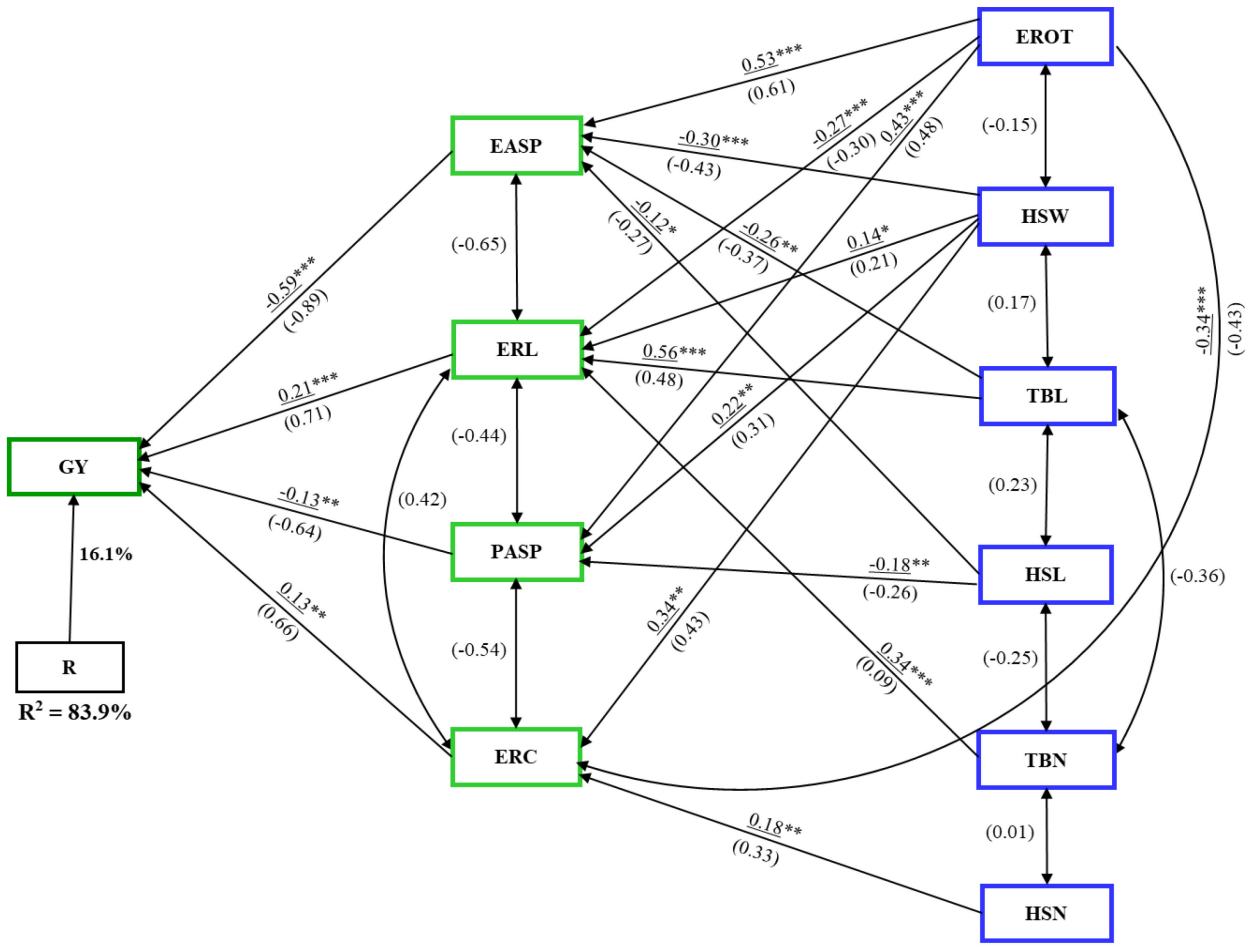
Path analysis model illustrating the causal relationships between grain yield (GY), husk, ear, tassel, and agronomic traits under combined artificial *Striga* infestation and rainfed conditions. Underlined values represent direct path coefficients, while values in parenthesis indicate correlation coefficients. Single-arrow lines represent path coefficients, while double-arrow lines denote correlation coefficients. EASP, ear aspect; ERC, ear circumference; ERL, ear length; EROT, Percentage of rotten ears; HSL, husk length; HSN, husk number; HSW, husk width; PASP, plant aspect; TBL, tassel branch length; TBN, tassel branch number. *, **, *** Significant at the 0.05, 0.01, and 0.001 probability levels, respectively.

In the combined analysis across artificial *Striga* infestation and rainfed conditions, the first-order traits – EASP, ERL, ERC, and PASP – explained 84% of the variability in GY (**Figure 6**). ERL and ERC had positive direct path coefficients (0.21 and 0.13, respectively), while EASP and PASP showed negative direct path coefficients (–0.59 and –0.13, respectively) with GY. Similar to the analysis under rainfed conditions, only first- and second-order traits were identified. Second-order traits EROT, HSW, TBL, and TBN influenced GY indirectly through ERL. TBL and EROT had the largest indirect effects (0.56 and 0.53, respectively).

## Discussion

Maize grain yield is influenced by the partitioning of assimilates between vegetative and reproductive tissues, particularly during critical growth phases such as flowering and grain-filling. Improved yields in temperate maize have been attributed to changes in plant and ear morphology, an extended grain-filling period, and tolerance to both biotic and abiotic stresses (Duvick, 2005; Fischer and Edmeades, 2010; Lauer et al., 2012; Egli, 2015). Among the traits associated with increased yields are those related to the ear and tassel. A limited number of studies have explored the variability in some of the plant and ear morphological traits that are crucial for grain yield in tropical maize (Guei and Wassom, 1996; Sofi, 2007; Sorsa et al., 2023). This study examined the genetic variation and inheritance of husk, ear, and tassel traits in mid-altitude adapted tropical maize lines under different management conditions.

### Genetic variability and heritability

Breeding programs rely on genetic variability to make selections leading to genetic improvements in traits of interest. The significant genotypic differences observed among the parental lines and hybrids for most of the traits indicate sufficient genetic variability in the germplasm used in this study to enable progress through selection for these traits. This result is in agreement with findings from other studies on maize under varying stress and non-stress conditions (Betrán et al., 2003; Makumbi et al., 2018; Badu-Apraku et al., 2020). Furthermore, genetic variances were significant and generally exceeded the magnitude of G × E interaction variances for most traits, consistent with findings from studies on *Striga*-resistant germplasm with diverse genetic backgrounds (Menkir and Kling, 2007; Kimutai et al., 2024). The significant G × E interactions for most traits suggest variability in genotype responses, likely influenced by differing climatic conditions across the seasons of evaluation. Similar results have been reported for various traits under *Striga* infestation across seasons (Menkir et al., 2012; Makumbi et al., 2015; Kanampiu et al., 2018; Kimutai et al., 2024), as well as under rainfed and managed stress conditions (Bolaños, 1995; Makumbi et al., 2011, 2018; Mageto et al., 2017; Worku et al., 2016), underscoring the importance of multi-environment or multi-year evaluations for identifying stable genotypes. Conversely, the non-significant G × E interaction for some traits (HSL and HSW) under rainfed conditions suggests these traits were stable across environments, and that genotype evaluation for these traits can rely primarily on genetic differences, with minimal environmental influence, potentially reducing the need for extensive multi-environment testing.

Broad-sense heritability estimates for husk, ear, and tassel traits in this study were predominantly high for both hybrids and lines across all management conditions, except for moderate estimates for ERC, HSL, and HSW under managed drought stress. These results suggested that much of the observed variability is due to genetic factors, indicating strong potential for genetic gains through selection. Consequently, narrow-sense heritability estimates for these traits is also expected to be high (Falconer and Mackay, 1996). Brewbaker (2015) reported high narrow-sense heritability for TBN in some biparental populations of temperate maize, whereas Guei and Wassom (1996) found low narrow-sense heritability for TBN in tropical maize. Sorsa et al. (2023), reported low to moderate broad-sense heritability estimates for ERL and ear diameter in tropical maize. Multiple studies have reported moderate to high heritability estimates for ear traits such as ERL and ear diameter (Han and Hallauer, 1989; Yu et al., 2020; Yang et al., 2021; Wang J. et al., 2023; Chang et al., 2024), tassel traits like TBN and TBL (Mock and Schuetz, 1974; Schuetz and Mock, 1978; Geraldi et al., 1985; Upadyayula et al., 2006; Yi et al., 2019; Zeng et al., 2022), and husk traits including HSL, HSN, and HSW (Zhou et al., 2020; Zhang et al., 2021, 2022) in temperate maize.

### Relative importance of additive and nonadditive effects, and combining ability

The significant GCA and SCA suggested that both additive and nonadditive genetic effects influenced the inheritance of husk, ear, and tassel traits across all contrasting environments. This result suggested that simple selection methods could be effective for improving traits such as ERC and TBN. Previous inheritance studies in temperate and tropical maize have reported variation in the relative importance of additive and nonadditive genetic effects for these traits. For TBN, our results are consistent with the findings in several studies that highlighted the importance of additive genetic effects in temperate and tropical maize (Mock and Schuetz, 1974; Schuetz and Mock, 1978; Guei and Wassom, 1996; Nardino et al., 2016; Onejeme et al., 2020; Kamara et al., 2020). Husk number is primarily under the influence of additive genetic effects (Brewbaker and Kim, 1979). Similarly, both additive and nonadditive effects were reported to influence ear length, diameter, and circumference in maize of different adaptations (Dhillon and Singh, 1977; Onejeme et al., 2020; Kamara et al., 2020). Kamara et al. (2014) reported the importance of non-additive gene action over additive gene action for ear length and diameter.

In this study, the GCA sums of squares predominated the SCA sums of squares for husk, ear, and tassel traits across all management conditions. These findings indicate that additive gene action was predominant in the inheritance of ear, husk, and tassel traits across all management conditions. This is consistent with previous studies that reported significant contribution of additive gene action to traits such as ERL and ear diameter (Han and Hallauer, 1989; Fan et al., 2008; Kamara et al., 2020), husk number (Brewbaker and Kim, 1979), and TBN (Betrán and Hallauer, 1996; Brewbaker, 2015). Wolf and Hallauer (1997) reported that epistatic effects were important for ERL in temperate maize. The study revealed significant GCA_m_/sets × E, GCA_f_/sets × E, and SCA/sets × E interactions for most traits under artificial *Striga* infestation, rainfed, and managed drought stress conditions, indicating that the GCA effects and hybrid performance (SCA) varied across environments. Similar results have been reported for various agronomic traits under *Striga* infestation, low N, and managed drought stress conditions (Makumbi et al., 2011; Badu-Apraku et al., 2016; Njeri et al., 2017).

The GCA effects for husk, ear, and tassel traits varied across contrasting environments. The consistently significant positive GCA (GCA_f_ and GCA_m_) effects for HSL expressed by six inbred lines (CML610A, CKDHL171092, CKDHL171119, CKDHL171527, CKL17531 and TZSTR189) under both artificial Striga infestation and rainfed conditions, indicated that these inbreds possess favorable alleles for longer husks. Notably, CML610A also exhibited significant GCA effects for HSL under managed drought stress, indicating consistency of its GCA effects across environments. Nine inbred lines showed significant positive GCA_f_ and GCA_m_ effects for HSW, suggesting that these lines carry favorable alleles for increased HSW. Interestingly, CML610A, which had positive GCA effects for HSL, also showed positive GCA effects for HSW, indicating that this line carries good favorable allele combinations that can be exploited in breeding programs. The results under artificial Striga infestation showed that 16 lines carried favorable alleles for increased HSN (significant positive GCAf or GCAm effects), while others exhibited significant negative GCAf or GCAm effects for HSN. Similar patterns were observed under rainfed and managed drought stress conditions. These findings suggest that both maternal and paternal effects influence inheritance of HSN, implying that the choice of a line as either the female or male parent could impact the expression of husk number in a hybrid.

Husks in maize serve various functions, from contributing to photosynthesis to protecting developing kernels from birds, insect pests, and diseases. The GCA effects observed in this study suggest that this germplasm offers favorable alleles for breeding maize suited to different needs. For instance, increased husk numbers have been linked to reduced damage from fall armyworm (FAW) and corn earworm (CEW) (Brewbaker and Kim, 1979), making lines with favorable GCA effects for increased HSN valuable for breeding programs. However, shorter husk leaves may increase the vulnerability of kernels to insect and bird damage. Inbred lines with favorable GCA effects for longer husks can be used to develop hybrids with better husk coverage to protect kernels and avoid open tips. An interesting area for future research would be to evaluate the impact of husk traits such as number, tightness, and length on FAW ear damage in sub-Saharan Africa (SSA). Additionally, husk tightness has been identified as a barrier to Fusarium growth (Warfield and Davis, 1996; Cao et al., 2014) and aflatoxin contamination in maize (Barry et al., 1986). While increased husk number may be beneficial in some environments, fewer husks are preferred in temperate regions for faster drying (Troyer and Ambrose, 1971), and suitability for mechanical harvesting.

In this study, some inbred lines exhibited significant positive GCA_f_ and GCA_m_ effects for ERC and ERL artificial *Striga* infestation, suggesting that these inbred lines possess favorable alleles for larger and longer ears. The six lines with significant positive GCA_f_ and GCA_m_ effects for ERL (TEISTR1156, CKDHL171119, CKDHL171267, CKDHL171357, CKL17535, and CKL17571) have a breeding history of *Striga* resistance, with one line among these (TEISTR1156) showing promising results when tested in hybrid combination under artificial *Striga* infestation (Menkir et al., 2012). The putative *Striga* resistance in these lines may have contributed to their favorable ear characteristics under stress conditions caused by *Striga* infestation. Additionally, four lines expressed consistent significant positive GCA_f_ and GCA_m_ effects for ERL under both rainfed and artificial *Striga* infestation conditions, indicating that these lines possess favorable alleles for these traits under both stressed and non-stressed conditions. Three inbred lines (CKDHL171267, CKDHL171357, and CKL17535) exhibited consistently significant positive GCA_f_ and GCA_m_ effects for ERL under both artificial *Striga* infestation and managed drought conditions, indicating their genetic value in maintaining ear length under biotic and abiotic stress conditions. Ear length and ear diameter are some of the critical grain yield components in maize, and traits like these can be used as indirect selection criteria for improved grain yield. Inbred lines with desirable GCA effects for increased ear length identified in this study are potential candidates for use in a selection program aimed at improving ear length in mid-altitude tropical maize germplasm.

Grain yield results from both dry matter accumulation and its partitioning to the grain at physiological maturity, with the latter being a function of kernels per plant and weight per kernel (Tollenaar and Lee, 2006; Lee and Tollenaar, 2007). Grain yield limitations in maize other than those induced by biotic and abiotic stresses can be examined by considering the availability of assimilates to the developing grain (source), and the capacity of the grains to store the assimilates (sink) (Tollenaar, 1977). Sink size can be a limiting factor in maize, but it can be overcome by increasing ear size (Egli, 2015). The two ear traits (ERL and ERC) described herein are important in determination of kernels per plant and therefore contribute to the size of the sink, and ultimately grain yield in maize. Therefore, breeding efforts to develop genotypes with longer or larger ears can lead to yield increase. In-depth studies using a targeted number of hybrids developed from inbred lines with contrasting GCA effects for ear traits identified in this study could enhance our understanding of source-sink relationships in this germplasm. This approach would be especially valuable under *Striga* infestation, where the balance between assimilate production and utilization is affected. Previous research involving a limited number of hybrids has successfully explored such dynamics (e.g. Rajcan and Tollenaar, 1999; Tollenaar and Daynard, 1978, 1982; Uhart and Andrade, 1991), providing a foundation for applying similar methodologies to this unique set of germplasm. Furthermore, genomic approaches could be employed to investigate source-sink relationships under artificial *Striga* infestation, offering deeper insights into the underlying mechanisms (e.g. Welcker et al., 2007; Kumar et al., 2023).

In this study, 10 inbred lines exhibited significant negative GCA_f_ effects for TBL, and 13 lines showed significant negative GCA_m_ effects for TBN under artificial *Striga* infestation, indicating favorable alleles for reduced tassel branch length and number. Seven inbred lines showed GCA effects for reduced TBN under both rainfed and artificial *Striga* infestation, reflecting their consistency in expression of favorable alleles for a lower TBN across a range of environments. Studies have shown that reduced TBN is associated with increased grain yield primarily due to better light interception and reduced competition for assimilates between the tassel and the ear (Hunter et al., 1969; Mock and Pearce, 1975; Sangoi, 2001). Our results revealed that there are several inbred lines with potential for reduced TBN, which could lead to higher grain yield when used in hybrid combinations. These lines would be valuable for breeding programs, especially if they also combine low TBN with multiple stress tolerance, a key requirement for maize breeding in SSA. A study by Lauer et al. (2012) reported that TBN in US inbred lines has steadily decreased from an average of 12 in the 1930s to an average of 6 in the 2000s, a trend attributed to systematic selection for smaller tassel size.

### Genetic correlations, heterosis, and path analysis

Genetic correlations between traits provide insights into breeding strategies. The results of this study revealed significant positive genetic correlations between ear traits (ERL and ERC) and grain yield, indicating that selecting for increased ear length and circumference can enhance grain yield. The high heritability estimates for these two traits combined with their strong genetic correlation with grain yield, suggest that they could serve as effective indirect selection criteria for grain yield improvement. Conversely, grain yield showed a negative correlation with TBN under *Striga* infestation, but a positive correlation with TBN under rainfed conditions. In a study with tropical × temperate hybrids, Ndou et al. (2021) did not find a significant correlation between GY and TBN. This suggests that the genes controlling the two traits are differentially affected by different physiological mechanisms depending on the environment (Falconer and Mackay, 1996). While genetic correlations are typically population specific, a previous study also reported a negative correlation between TBN and grain yield (Geraldi et al., 1985). Interestingly, no strong correlation was found between TBN and husk traits under rainfed conditions, which contrasts with a study by Brewbaker (2015) which reported a strong correlation between TBN and HSN in tropical maize.

Our results revealed that heterosis for ERL and ERC was more expressed under managed drought stress compared to *Striga* infestation. This aligns with previous studies (Betrán et al., 2003; Makumbi et al., 2011; 2018) in which greater magnitude of heterosis under managed drought stress than other stress conditions was reported. The heterosis estimate for ERL under *Striga* infestation in this study was comparable to that reported by Yu et al. (2020). Since heterosis is generally more pronounced in ERL (Troyer and Ambrose, 1971), breeding programs should use lines with desirable GCA effects for ERL such as those identified in this study to develop hybrids that can express significant heterosis for ERL. Under managed drought stress, inbred line performance is more severely impacted than that of hybrids, resulting in larger heterosis estimates.

Path coefficient analysis revealed that EASP had the largest negative direct path coefficient, while ERL and ERC consistently showed positive direct path coefficient with GY across all conditions. This suggests that EASP, along with ear traits ERL and ERC, are critical when evaluating GY potential of genotypes under both artificial *Striga* infestation and rainfed conditions. These traits should be integrated into a selection index to facilitate hybrid advancement (e.g., Badu-Apraku et al., 2014; Makumbi et al., 2018; Crispim-Filho et al., 2020). In particular, a base index that incorporates trait-specific heritability estimates would be most effective (e.g. Smith et al., 1981; Makumbi et al., 2018). Additionally, these traits can serve as indirect selection criteria to improve GY. A high and significant genetic correlation between GY and these traits further supports this conclusion. Similarly, Silveira et al. (2021) proposed tassel traits as indirect selection criteria. While measuring ERL and ERC can be time-consuming, the adoption of new high-throughput ear phenotyping techniques (Miller et al., 2017; Makanza et al., 2018; Oury et al., 2022; Liu et al., 2023; Wang J. et al., 2023) is expected to significantly enhance the speed and efficiency of capturing these traits in breeding programs.

### Genetic improvement of ear, husk, and tassel traits in tropical maize

The genetic improvement of tropical maize in sub-Saharan Africa and Latin America has primarily focused on biotic and abiotic stress tolerance, as well as improved grain yield, with notable success (Bänziger et al., 2006; Badu-Apraku et al., 2013; Prasanna et al., 2020; 2021). This work targeted selection for traits related to stress tolerance, such as ears per plant, reduced anthesis-silking interval, resistance to stalk and root lodging, and resistance to foliar and virus diseases, alongside improving final grain yield in the hybrids and synthetics. However, there was limited focus on selection for individual traits that enhance efficiency in assimilate utilization and ultimately increase grain yield. Such traits include but are not limited to reduced tassel size and branch number, ear length and diameter, kernel rows per ear, husk tightness and extension length, and leaf architecture. In contrast, temperate maize breeding programs have consistently selected for these traits, resulting in inbred lines with better plant architecture and higher grain yield *per se* and in hybrid combinations (Russell, 1991; Duvick, 2005; Wang et al., 2011; Lauer et al., 2012; Liu et al., 2021). Earlier efforts to systematically improve traits like reduced TBN in tropical maize were reported (Fischer et al., 1987; Chapman and Edmeades, 1999). We believe more attention should be given to systematically improving these traits in tropical maize, particularly in SSA, to develop new inbred lines that efficiently convert nutrients into higher grain yields. Selection for these traits has been successful in temperate regions (Fakorede and Mock, 1978; Schuetz and Mock, 1978; Cortez-Mendoza and Hallauer, 1979; Lopez-Reynoso and Hallauer, 1998). The improvement of these traits in tropical maize could be achieved through systematic introgression of ex-PVP temperate germplasm that carries favorable alleles for some of these traits (e.g., Abadassi and Hervé, 2000; Cupertino-Rodrigues et al., 2020; Dao et al., 2020; Musundire et al., 2021; Ndou et al., 2021). A reduction in TBN in tropical × temperate maize crosses has been reported (Ndou et al., 2021). A promising breeding strategy could involve developing biparental populations by crossing lines with reduced TBN and increased ERL and ERC identified in this study, with ex-PVP lines for DH induction. However, this approach must carefully consider the alignment of heterotic patterns between tropical and temperate maize. Faster and more efficient identification of inbred lines with favorable allele combinations for traits such TBN, ERL, ERC, and HSN can be achieved through marker-assisted selection (MAS), following fine mapping of quantitative trait loci (QTL) reported in several studies (Huo et al., 2016; Khatun et al., 2022; Ruidong et al., 2023; Wang X. et al., 2023; Zeng et al., 2022; Xiao et al., 2016; Zhou et al., 2020; Zhu et al., 2018).

## Conclusions

The study revealed that additive gene action predominated in the inheritance of ear, husk, and tassel traits across all management conditions. Inbred lines with consistently favorable GCA effects for increased ERL and ERC, along with favorable GCA effects for reduced TBN and TBL, were identified, indicating their suitability for hybrid development, and the formation of biparental breeding populations. Heterosis estimates were higher for ear and tassel traits under stress conditions. Genetic correlations between grain yield and ERL and ERC were strong and positive. Path analysis revealed ERL, ERC, and EASP were the first-order traits most strongly correlated with grain yield, highlighting their value for inclusion in a selection index. The broad-sense heritability estimates for husk, ear, and tassel traits were mostly high, indicating the potential for significant genetic gains from selection for these traits. The inbred lines evaluated in this study exhibited higher average TBN compared to US inbred lines. Tropical maize inbred lines could be improved for the key grain yield component traits through the introgression of temperate germplasm to develop more efficient and higher yielding inbred lines.

## Data Availability

The original contributions presented in the study are included in the article/**Supplementary Material**. Further inquiries can be directed to the corresponding author.

## Supplementary material

Supplementary Table 1 List, origin, and set information of 30 inbred lines used in a North Carolina Design II mating scheme to generate 150 hybrids.

Supplementary Table 2 Test location characteristics (coordinates, elevation, rainfall, and temperature) in 2020 and 2021.

Supplementary Table 3 Percentage of total genotypic sum of squares contribution for ear traits, plant aspect and tassel traits of 150 hybrids attributable to GCA-males (GCA_m_), GCA-females (GCA_f_), and SCA across managed drought stress conditions, 2020-2021.

Supplementary Table 4 Estimates of general combining ability effects of females (GCA_f_) and males (GCA_m_) of 30 tropical maize lines for plant and ear traits evaluated under artificial *Striga* infestation, rainfed, and managed drought stress conditions. **^a^** BHC, Bad husk cover; EASP, Ear aspect; EROT, Percentage of rotten ears; PASP, Plant aspect.

Supplementary Table 5 Summary of mean squares from combined ANOVA, descriptive statistics, and broad-sense heritability estimates for ear and tassel traits of inbred lines under different management conditions in Kenya (2020–2021). ^a^BHC, Bad husk cover; EASP, Ear aspect; ERL, Ear length; ERC, Ear circumference; EROT, Percentage of rotten ears; HSL, Husk length; HSN, Husk number; HSW, Husk width; PASP, Plant aspect; TBL, Tassel branch length; TBN, Tassel branch number.
