# Supplementary material for: Genetic analysis of ear, husk, and tassel traits in tropical maize under diverse environments
Source: Front Plant Sci. 2025 Aug 11;16:1618054. doi: 10.3389/fpls.2025.1618054 (PMC12375579; doi:10.3389/fpls.2025.1618054)
Supplement: Supplementary Table 1 — List, origin, and set information of 30 inbred lines used in a North Carolina Design II mating scheme to generate 150 hybrids. [file Table1.docx]

**SUPPLEMENTARY TABLE 1**. List, origin, and set information of 30 inbred lines used in a North Carolina Design II mating scheme to generate 150 hybrids.

| Line | Name | Type | Origin | Set | Female Set | Male Set |
| --- | --- | --- | --- | --- | --- | --- |
| 1 | TEISTR1156 | Pedigree | IITA | 1 | 5 | 4 |
| 2 | TEISTR1157 | Pedigree | IITA | 1 | 5 | 4 |
| 3 | TEISTR1158 | Pedigree | IITA | 1 | 5 | 4 |
| 4 | TEISTR1159 | Pedigree | IITA | 1 | 5 | 4 |
| 5 | TZSTR189 | Pedigree | IITA | 1 | 5 | 4 |
| 6 | CKDHL17888 | Doubled haploid | CIMMYT | 2 | 4 | 3 |
| 7 | CKDHL17961 | Doubled haploid | CIMMYT | 2 | 4 | 3 |
| 8 | CKDHL171092 | Doubled haploid | CIMMYT | 2 | 4 | 3 |
| 9 | CKDHL171119 | Doubled haploid | CIMMYT | 2 | 4 | 3 |
| 10 | CKDHL171162 | Doubled haploid | CIMMYT | 2 | 4 | 3 |
| 11 | CKDHL171267 | Doubled haploid | CIMMYT | 3 | 3 | 5 |
| 12 | CKDHL171357 | Doubled haploid | CIMMYT | 3 | 3 | 5 |
| 13 | CKDHL171514 | Doubled haploid | CIMMYT | 3 | 3 | 5 |
| 14 | CKDHL171520 | Doubled haploid | CIMMYT | 3 | 3 | 5 |
| 15 | CKDHL171527 | Doubled haploid | CIMMYT | 3 | 3 | 5 |
| 16 | CKDHL171564 | Doubled haploid | CIMMYT | 4 | 1 | 2 |
| 17 | CKL17535 | Pedigree | CIMMYT | 4 | 1 | 2 |
| 18 | CKL17571 | Pedigree | CIMMYT | 4 | 1 | 2 |
| 19 | CKL17604 | Pedigree | CIMMYT | 4 | 1 | 2 |
| 20 | CKL17611 | Pedigree | CIMMYT | 4 | 1 | 2 |
| 21 | CKL17508 | Pedigree | CIMMYT | 5 | 2 | 6 |
| 22 | CKL17513 | Pedigree | CIMMYT | 5 | 2 | 6 |
| 23 | CKL17517 | Pedigree | CIMMYT | 5 | 2 | 6 |
| 24 | CKL17531 | Pedigree | CIMMYT | 5 | 2 | 6 |
| 25 | CKL17650 | Pedigree | CIMMYT | 5 | 2 | 6 |
| 26 | CKL17719 | Pedigree | CIMMYT | 6 | 6 | 1 |
| 27 | CML610A | Pedigree | CIMMYT | 6 | 6 | 1 |
| 28 | CKL12128 | Pedigree | CIMMYT | 6 | 6 | 1 |
| 29 | CML543 | Pedigree | CIMMYT | 6 | 6 | 1 |
| 30 | CML312 | Pedigree | CIMMYT | 6 | 6 | 1 |

**SUPPLEMENTARY TABLE 2**. Test location characteristics (coordinates, elevation, rainfall, and temperature) in 2020 and 2021.

| Location | Latitude | Longitude | Elevation (m asl) | Year | Annual rainfall (mm) | Temperature (^o^C) | | Management |
| --- | --- | --- | --- | --- | --- | --- | --- | --- |
|  |  |  |  |  |  | Max | Min |  |
| Kibos | 0⁰2’S | 34⁰48E | 1193 | 2020 | 1563.6 | 27.9 | 14.5 | Artificial *Striga* infestation |
|  |  |  |  | 2021 | 1411.1 | 29.3 | 14.8 |  |
| Alupe | 0⁰30’N | 34⁰7E | 1250 | 2020 | 1265.4 | 30.5 | 12.8 | Artificial *Striga* infestation |
|  |  |  |  | 2021 | 1098.0 | 31.7 | 13.3 |  |
| Siaya | 03⁰10’N | 34⁰17E | 1288 | 2020 | 1761.8 | 30.5 | 17.2 | Artificial *Striga* infestation |
|  |  |  |  | 2021 | 1754.5 | 31.4 | 17.2 |  |
| Kakamega | 0^o^16'N | 34^o^49'E | 1585 | 2020 | 2414.6 | 26.8 | 10.3 | Rainfed conditions |
|  |  |  |  | 2021 | 1943.9 | 28.3 | 10.9 |  |
| Kiboko | 2^o^15'S | 37^o^75'E | 975 | 2020 | 630.0 | 33.8 | 10.5 | Managed drought stress |
|  |  |  |  | 2021 | 438.2 | 33.9 | 11.1 |  |

**SUPPLEMENTARY TABLE 3**. Percentage of total genotypic sum of squares contribution for ear traits, plant aspect and tassel traits of 150 hybrids attributable to GCA-males (GCA_m_), GCA-females (GCA_f_), and SCA across managed drought stress conditions, 2020-2021.

|  | **Managed drought stress** | | | | | |
| --- | --- | --- | --- | --- | --- | --- |
| **Trait** | GCA_m_ | | GCA_f_ | | SCA | |
| Husk length | 33.54 | 23.85 | | 42.61 | |  |
| Husk width | 28.39 | 33.66 | | 37.95 | |  |
| Husk number | 38.42 | 40.09 | | 21.49 | |  |
| Ear circumference | 26.76 | 32.00 | | 41.25 | |  |
| Ear length | 31.80 | 26.85 | | 41.36 | |  |
| Ear aspect | 21.60 | 30.48 | | 47.92 | |  |

**SUPPLEMENTARY TABLE 4**. Estimates of general combining ability effects of females (GCA_f_) and males (GCA_m_) of 30 tropical maize lines for plant and ear traits evaluated under artificial *Striga* infestation, rainfed, and managed drought stress conditions.

|  | Artificial *Striga* infestation | | | | | | | | Rainfed conditions | | | | | | | | Managed drought | |
| --- | --- | --- | --- | --- | --- | --- | --- | --- | --- | --- | --- | --- | --- | --- | --- | --- | --- | --- |
|  | **BHC^a^** | | **EROT** | | **EASP** | | **PASP** | | **BHC** | | **EROT** | | **EASP** | | **PASP** | | **EASP** | |
| **Name** | **GCA_f_** | **GCA_m_** | **GCA_f_** | **GCA_m_** | **GCA_f_** | **GCA_m_** | **GCA_f_** | **GCA_m_** | **GCA_f_** | **GCA_m_** | **GCA_f_** | **GCA_m_** | **GCA_f_** | **GCA_m_** | **GCA_f_** | **GCA_m_** | **GCA_f_** | **GCA_m_** |
| TEISTR1156 | 6.49*** | 5.00** | -0.56 | -0.25 | -0.12* | -0.19** | -0.04 | -0.07 | 1.66 | 2.97 | -2.67 | -5.41* | -0.25** | -0.17* | -0.32** | -0.07 | -0.19 | -0.09 |
| TEISTR1157 | 0.91 | -0.09 | -0.49 | -0.26 | -0.08 | 0.06 | -0.14* | 0.15** | 1.65 | 0.46 | -5.23* | -9.14** | -0.18 | -0.17* | -0.19* | -0.17 | 0.01 | -0.04 |
| TEISTR1158 | -3.39* | -1.22 | -0.40 | 0.59 | 0.11 | 0.00 | 0.03 | -0.18** | 1.95 | -2.94 | -1.60 | -1.20 | 0.17* | 0.08 | 0.29** | 0.16 | 0.06 | 0.14 |
| TEISTR1159 | -2.36 | -2.00 | 1.23* | -0.48 | 0.10 | 0.14* | 0.19** | 0.15** | -2.33 | -2.02 | 5.81* | 11.16*** | 0.14 | 0.27** | 0.06 | 0.01 | -0.02 | 0.03 |
| TZSTR189 | -1.64 | -1.68 | 0.22 | 0.41 | -0.02 | -0.01 | -0.04 | -0.05 | -2.93 | 1.53 | 3.70 | 4.58 | 0.12 | -0.02 | 0.16 | 0.06 | 0.14 | -0.04 |
| CKDHL17888 | -0.18 | 2.61 | -0.22 | 0.57 | 0.13* | 0.11 | -0.03 | -0.01 | 0.84 | 7.10 | 3.53 | 6.17* | 0.26** | 0.35*** | 0.04 | 0.13 | -0.01 | 0.04 |
| CKDHL17961 | 0.73 | 0.98 | -0.87 | 0.32 | 0.06 | -0.02 | 0.15** | -0.06 | -0.15 | 0.49 | 2.64 | 5.30* | 0.18* | 0.17* | 0.09 | 0.01 | 0.36** | 0.06 |
| CKDHL171092 | -2.38 | -6.46*** | 0.52 | -0.57 | -0.16* | -0.20** | -0.05 | 0.14* | -2.32 | -6.86 | 1.43 | -6.84* | -0.19* | -0.38*** | -0.02 | -0.09 | -0.32* | 0.04 |
| CKDHL171119 | -1.33 | -2.18 | -0.37 | -0.99 | -0.27*** | -0.27*** | -0.03 | -0.06 | -2.97 | -4.92 | -9.08** | -3.57 | -0.30** | -0.28** | -0.14 | 0.03 | -0.27* | -0.14 |
| CKDHL171162 | 3.15* | 5.06** | 0.94 | 0.66 | 0.25*** | 0.38*** | -0.05 | -0.01 | 4.59 | 4.19 | 1.48 | -1.06 | 0.06 | 0.15* | 0.04 | -0.09 | 0.24* | -0.01 |
| CKDHL171267 | 3.10* | 2.27 | 1.87** | 1.41* | 0.04 | -0.06 | 0.06 | 0.01 | 0.32 | 0.3 | -1.16 | 6.23* | -0.13 | -0.03 | 0.01 | 0.11 | -0.11 | 0.09 |
| CKDHL171357 | 0.63 | -0.15 | -0.62 | -0.72 | -0.02 | -0.01 | 0.09* | 0.04 | -0.79 | 4.21 | 1.85 | 3.75 | 0.13 | 0.12 | 0.16 | 0.06 | -0.31* | -0.04 |
| CKDHL171514 | 6.16*** | 1.97 | -0.19 | -1.39* | 0.04 | 0.06 | -0.06 | 0.01 | 4.87 | 0.72 | 4.87 | -5.18 | 0.09 | 0.07 | 0.01 | 0.06 | 0.17 | 0.09 |
| CKDHL171520 | -5.09** | -0.72 | -0.82 | 0.32 | 0.04 | 0.15* | 0.06 | -0.02 | -0.09 | -2.84 | 1.18 | 5.00 | -0.03 | 0.09 | -0.12 | 0.04 | -0.06 | 0.06 |
| CKDHL171527 | -4.80* | -3.37* | -0.25 | 0.38 | -0.10 | -0.13* | -0.16** | -0.04 | -4.31 | -2.39 | -6.74* | -9.81** | -0.05 | -0.25** | -0.06 | -0.27** | 0.32* | -0.19 |
| CKDHL171564 | 5.96*** | 5.50** | 0.65 | 0.22 | 0.49*** | 0.28*** | 0.16** | 0.12* | 7.99* | 5.34 | 7.06* | -0.76 | 0.38*** | 0.31*** | 0.07 | -0.05 | 0.33* | 0.23* |
| CKL17535 | 10.86*** | 14.88*** | -0.49 | 0.81 | -0.20** | -0.08 | -0.09 | -0.08 | 3.91 | 12.51** | 4.38 | 3.87 | 0.09 | 0.03 | 0.15 | 0.13 | -0.19 | -0.22 |
| CKL17571 | -0.59 | 0.88 | 0.34 | -0.39 | -0.15* | -0.26*** | -0.12* | -0.14* | -1.12 | -5.77 | -2.36 | -0.51 | -0.26** | -0.30** | -0.15 | -0.18* | -0.15 | -0.09 |
| CKL17604 | -8.90*** | -4.32* | -0.24 | -0.04 | -0.05 | 0.07 | 0.06 | 0.01 | -1.75 | -0.74 | -6.66* | -0.94 | -0.19* | -0.09 | -0.13 | -0.10 | 0.03 | -0.14 |
| CKL17611 | -7.32*** | -16.95*** | -0.27 | -0.61 | -0.08 | -0.01 | 0.00 | 0.09 | -9.03* | -11.33* | -2.42 | -1.66 | -0.01 | 0.05 | 0.05 | 0.20* | -0.02 | 0.23* |
| CKL17508 | -1.49 | -5.25** | -0.07 | -0.60 | -0.10 | -0.27*** | -0.01 | -0.02 | -7.85* | -4.99 | 0.76 | 0.95 | -0.07 | 0.06 | 0.00 | 0.12 | -0.05 | 0.11 |
| CKL17513 | 11.69*** | 13.95*** | -0.60 | 0.84 | 0.03 | 0.18** | -0.01 | 0.08 | 11.42* | 21.53*** | -3.86 | -2.2 | -0.09 | 0.09 | 0.05 | -0.03 | -0.07 | 0.03 |
| CKL17517 | 0.84 | 0.85 | -0.72 | -0.13 | 0.07 | 0.01 | 0.17** | -0.02 | 2.68 | 3.98 | 0.86 | 2.53 | 0.18* | 0.09 | -0.25* | -0.06 | 0.38** | 0.26* |
| CKL17531 | -7.60*** | -9.16*** | 0.67 | 1.23* | -0.04 | 0.12* | -0.11* | -0.06 | -3.02 | -8.03* | 0.16 | 0.61 | -0.07 | -0.03 | 0.30** | 0.24* | -0.09 | -0.04 |
| CKL17650 | -3.44* | -0.39 | 0.70 | -1.34* | 0.04 | -0.04 | -0.04 | 0.01 | -3.24 | -12.49** | 2.09 | -1.88 | 0.05 | -0.21* | -0.10 | -0.28** | -0.17 | -0.35** |
| CKL17719 | 6.38*** | 8.12*** | -0.27 | 0.69 | -0.08 | 0.10 | 0.06 | 0.21*** | -0.65 | 12.79** | 5.88* | 4.38 | 0.31*** | 0.26** | 0.04 | 0.00 | 0.31* | 0.43** |
| CML610A | 2.09 | -0.75 | -0.94 | -0.8 | -0.11 | -0.08 | -0.06 | -0.07 | 2.32 | -3.53 | -5.78* | -3.19 | -0.31*** | -0.29** | -0.01 | -0.05 | -0.14 | -0.07 |
| CKL12128 | 4.10* | -0.31 | -0.14 | -0.05 | -0.17* | -0.23** | -0.07 | -0.02 | -3.17 | -0.95 | 2.36 | -0.69 | -0.09 | 0.14 | 0.17* | 0.02 | -0.22 | -0.32* |
| CML543 | -9.42*** | -4.34* | 1.01 | -1.04 | 0.20** | 0.05 | -0.12* | -0.12* | -1.17 | 1.49 | -5.10 | -1.01 | -0.21* | -0.14 | -0.28** | -0.07 | 0.01 | -0.17 |
| CML312 | -3.15* | -2.74 | 0.34 | 1.20* | 0.17* | 0.16* | 0.19** | 0.00 | 2.67 | -9.81* | 2.63 | 0.52 | 0.29** | 0.04 | 0.07 | 0.10 | 0.05 | 0.13 |
| SE | 1.48 | | 0.60 | | 0.06 | | 0.05 | | 3.91 | | 2.62 | | 0.08 | | 0.09 | | 0.12 | |

**^a^**BHC, Bad husk cover; EASP, Ear aspect; EROT, Percentage of rotten ears; PASP, Plant aspect.

**SUPPLEMENTARY TABLE 5**. Summary of mean squares from combined ANOVA, descriptive statistics, and broad-sense heritability estimates for ear and tassel traits of inbred lines under different management conditions in Kenya (2020‒2021).

| Trait | Environment (E) | Genotype (G) | G × E | Mean | Range | LSD_0.05_ | *H^2^* |
| --- | --- | --- | --- | --- | --- | --- | --- |
| Artificial *Striga* infestation | | | | | | | |
| ERC^a^ | 26.52*** | 5.64*** | 0.76*** | 11.69 | 10.82 ‒ 13.03 | 0.75 | 0.88 |
| ERL | 24.67*** | 11.44*** | 2.11*** | 12.85 | 11.15 ‒ 14.90 | 1.19 | 0.83 |
| HSL | 65.98*** | 11.85*** | 2.05** | 19.15 | 17.21 ‒ 21.02 | 1.15 | 0.85 |
| HSN | 39.89*** | 14.76*** | 1.56 | 10.18 | 8.04 ‒ 12.36 | 0.94 | 0.94 |
| HSW | 25.72*** | 11.64*** | 1.65* | 9.05 | 6.95 ‒ 11.53 | 1.03 | 0.88 |
| BHC | 11655.37*** | 686.49*** | 300.97 | 10.93 | 4.76 ‒ 25.09 | 12.98 | 0.56 |
| EASP | 8.38*** | 1.30*** | 0.33*** | 3.08 | 2.61 ‒ 3.80 | 0.44 | 0.77 |
| EROT | 2808.47*** | 406.73*** | 204.24** | 7.66 | 3.86 ‒ 17.08 | 8.92 | 0.50 |
| TBL | 9.36*** | 28.98*** | 2.49* | 14.66 | 10.74 ‒ 19.24 | 1.55 | 0.93 |
| TBN | 266.14*** | 111.65*** | 6.48** | 13.44 | 7.71 ‒ 24.01 | 2.45 | 0.95 |
| Rainfed conditions | | | | | | | |
| EASP | 18.02*** | 0.90*** | 0.19 | 3.40 | 2.78 ‒ 4.17 | 0.56 | 0.79 |
| PASP | 37.97*** | 0.43** | 0.46** | 3.39 | 3.39 ‒ 3.39 | 0.00 | 0.00 |
| TBL | 127.54*** | 11.22*** | 3.07 | 14.64 | 12.40 ‒ 16.89 | 2.25 | 0.72 |
| TBN | 343.57*** | 51.93*** | 3.50 | 11.86 | 5.87 ‒ 20.52 | 2.89 | 0.93 |
| Managed drought stress | | | | | | | |
| ERC | 1134.57*** | 0.96*** | 0.32** | 6.96 | 6.49 ‒ 7.88 | 0.68 | 0.67 |
| ERL | 9.99* | 11.75*** | 2.56 | 10.24 | 7.47 ‒ 13.41 | 2.06 | 0.80 |
| HSN | 45.08*** | 7.04*** | 1.15 | 10.62 | 8.21‒13.06 | 1.47 | 0.84 |
| EASP | 25.21*** | 1.20*** | 0.33 | 3.83 | 2.86 ‒ 4.41 | 0.72 | 0.73 |

^a^BHC, Bad husk cover; EASP, Ear aspect; ERL, Ear length; ERC, Ear circumference; EROT, Percentage of rotten ears; HSL, Husk length; HSN, Husk number; HSW, Husk width; PASP, Plant aspect; TBL, Tassel branch length; TBN, Tassel branch number.
